# Using multivariate partial least squares on fNIRS data to examine load-dependent brain-behaviour relationships in aging

**DOI:** 10.1371/journal.pone.0312109

**Published:** 2024-10-14

**Authors:** Claudia Gonzalez, Supreeta Ranchod, Mark Rakobowchuk

**Affiliations:** 1 Psychology Department, Faculty of Arts, Thompson Rivers University, Kamloops, British Columbia, Canada; 2 Biology Department, Faculty of Science, Thompson Rivers University, Kamloops, British Columbia, Canada; Duke University Medical Center: Duke University Hospital, UNITED STATES OF AMERICA

## Abstract

Researchers implementing non-invasive neuroimaging have reported distinct load-dependent brain activity patterns in older adults compared with younger adults. Although findings are mixed, these age-related patterns are often associated with compensatory mechanisms of cognitive decline even in the absence of direct comparisons between brain activity and cognitive performance. This study investigated the effects of cognitive load on brain-behavior relationships in younger and older adults using a data-driven, multivariate partial least squares (PLS) analysis of functional near-infrared spectroscopy (fNIRS) data. We measured bilateral prefrontal brain activity in 31 older and 27 younger adults while they performed single and dual 2-back tasks. Behavioral PLS analysis was used to determine relationships between performance metrics (reaction time and error rate) and brain oxygenation (HbO) and deoxygenation (HbR) patterns across groups and task loads. Results revealed significant age-group differences in brain-behavior relationships. In younger adults, increased brain activity (i.e., increased HbO and decreased HbR) was associated with faster reaction times and better accuracy in the single task, indicating sufficient neural capacity. Conversely, older adults showed a negative correlation between HbR and error rates in the single task; however, in the dual task, they demonstrated a positive relationship between HbO and performance, indicative of compensatory mechanisms under the higher cognitive load. Overall, older adults’ showed relationships with either HbR or HbO, but not both, indicating that the robustness of the relationship between brain activity and behavior varies across task load conditions. Our PLS approach revealed distinct load-dependent brain activity between age groups, providing further insight into neurocognitive aging patterns, such as compensatory mechanisms, by emphasizing the variability and complexity of brain-behavior relationships. Our findings also highlight the importance of considering task complexity and cognitive demands in interpreting age-related brain activity patterns.

## Introduction

Aging has long been associated with cognitive changes even in the absence of neuropathology [1, 2]. With the world-wide increase in the proportion of older adults [3] and recent attempts to develop novel treatments for neurocognitive diseases like Alzheimer’s disease (AD) [4], it is imperative to better understand the cognitive changes occurring in the aging brain. This knowledge may be useful in better predicting cognitive changes corresponding to healthy aging trajectories, distinct from decline that may progress into a neurodegenerative disease.

Research implementing non-invasive neuroimaging methods has led to four main neurocognitive aging theories [5] and correspond to i) *dedifferentiation* [6, 7]; ii) *brain maintenance* [8, 9]; iii) *neural compensation* [10–14]; and iv) *neural inefficiency* [15, 16]. These theories attempt to explain the relationship between age-related changes in functional brain activity patterns and cognition or a lack of differences as in the case of brain maintenance, that is, some older adults exhibit similarities in brain activity and cognitive performance compared with younger adults [5, 8, 17]. The dedifferentiation theory describes less distinct or selective brain activity patterns, involving activation and suppression of brain regions, typically associated with poorer cognition [5, 18]. Evidence for dedifferentiation has been demonstrated through tasks that elicit selective activation in young adults in response to stimuli, such as face or object perception in ventral visual cortex [18, 19]. However, some report that dedifferentiation can be beneficial to performance [20] and/or that its effects on performance may not vary significantly with age [20, 21]. Conversely, the neural inefficiency and compensatory views both describe increased brain activity in older adults compared with younger, but with differing cognitive outcomes: enhanced performance in the compensatory view [11–14], and either unchanged or diminished performance in the neural inefficiency view [5, 15, 16], that is, additional activity is either not useful or detrimental to cognitive performance.

The compensatory view remains predominant with more evidence showing that older adults exhibit increased brain activity compared with younger adults [11–14], mainly involving the prefrontal cortex (PFC), to compensate for age-related decline [10, 22]. Cabeza et al. [17] noted that in the past years, some studies characterized brain activity as compensatory without directly associating the additional activity with performance. Thus, to avoid the over-interpretation and distinguish compensation from inefficiency or dedifferentiation, Cabeza et al. [17] recommended that for increased brain activity to be compensatory it must correlate with better performance and/or be associated with some gap between neural resources and task demands. In line with these criteria, compensation has been typically examined by implementing tasks with distinct cognitive loads [23–25].

Increases in brain activity are observed with increasing cognitive load, followed by a decrease when cognitive limits are reached (the compensation related utilization of neural circuits hypothesis or CRUNCH by Reuter-Lorenz and Cappell [26]). However, previous reports suggest that older adults reach cognitive limits earlier at lower loads compared with younger adults, and at higher loads no compensation is observed leading to poorer performance [9, 17, 23, 27]. This early attenuation has been challenged by several studies using functional magnetic resonance imaging (fMRI) [25, 28, 29], electroencephalography (EEG) [30], and functional near-infrared spectroscopy (fNIRS) [31–33], which report increased brain activity in older adults at higher task loads. Furthermore, it is not clear whether the increase in brain activity at higher loads supports performance [30, 32, 34] or not [25, 28]. Thus, discrepancies exist regarding age-related and load-dependent activations in older adults, adding to the challenges of characterizing neurocognitive aging.

Although these discrepancies may be dependent on different imaging techniques and/or task demands, they may also result from limited methodological adoptions. For example, age-group averaging of brain activity correlated with averaged performance may be initially useful to identify age-related differences but do not account for individual variation and are thus limited [23, 32]. Additionally, some reports investigating load-dependent activation in distinct groups that included direct brain-behaviour correlations have also used univariate approaches that result in multiple comparisons, increasing the risk of false discoveries; or to ameliorate this, some researchers have averaged over large brain regions and/or distinct load conditions, thus forcing researcher-defined constraints on results [35–37].

Morcom and Henson [38] examined prefrontal activity during memory tasks using fMRI in a large sample spanning 19 to 88 years of age. Although their univariate analysis revealed increased brain activity that would correspond with compensation, their multivariate analysis demonstrated that increased activity was more consistent with reduced efficiency or dedifferentiation [38]. Whilst univariate analyses are advantageous in identifying changes in brain activity of specific, discrete brain areas (e.g., voxels in fMRI or channels in fNIRS), multivariate approaches are better suited for establishing differences in functional brain activity and determining links with performance since these examine correlations or covariances of brain activity across brain areas, rather than considering brain areas or channels independent of each other, and result in better statistical power [39]. Additionally, multivariate analyses are data-driven approaches, not limited by researcher-defined constraints [38, 40], such as grouping across brain regions [35] or dividing older adults into high vs. low performing groups [41].

Multivariate partial least-squares (PLS) has long been used in neuroimaging [42] and is a robust method that can extract load-dependent changes in brain activity (i.e., task PLS) and evaluate brain activity and performance correlations (i.e., behaviour PLS) [43]. Recently, Meidenbauer et al. [40] examined the relationship between brain activity and N-back performance (i.e., accuracy) with increasing task loads in a young adult sample using behavioural PLS on fNIRS data. They showed that young adults’ brain activity was sensitive to cognitive load and differentially affected by performance across load conditions [40]. Similarly, Li et al. [44] examined fNIRS signal variability in an older adult population using PLS across task load (3 levels of N-back). They reported load-dependent changes in signal variability, specifically increased oxyhemoglobin variability at higher task loads [44]. Thus, applying PLS to fNIRS data was an effective way to examine significant correlations between brain activity and performance, without the constraints of univariate analyses. Furthermore, fNIRS is a non-invasive imaging technique similar to fMRI except it uses light emitting optodes at near-infrared wavelengths to determine changes in oxy (HbO) and deoxyhemoglobin (HbR) [45, 46] and has advantages over other techniques (e.g., low cost, larger sample sizes). Previously, researchers have demonstrated fNIRS sensitivity to cognitive load [35, 40, 47, 48] and age-related neurocognitive changes [49, 50]; however, there is a limited body of research examining the combined effects of load-dependent and age-related changes [27, 31, 33, 51] and few have implemented multivariate approaches combining age and load effects [40, 44].

Our primary aim was to examine the relationship between brain activity, measured using fNIRS, and cognitive performance across age-groups performing tasks with increasing cognitive load by implementing PLS, similar to Meidenbauer et al. [40], and robust methods of fNIRS data analyses [52, 53]. This multivariate data-driven approach enabled us to identify significant changes in brain activity related to distinct task loads, and make direct brain-behaviour correlations, as recommended by Cabeza et al. [17], to better characterize age-related brain activity patterns. We used previously reported data sets of older and younger adults in Ranchod et al. [32] in which age-related and load-dependent functional differences were observed using channel and region of interest (ROI) analyses. Cognitive load was increased from a single to a dual N-back task given that dual tasks impose greater processing demands (i.e., attentional and working memory demands for two simultaneous stimuli) and have been shown to elicit age-related differences in performance [54, 55]. Like Meidenbauer et al. [40], we report both HbO and HbR data, the latter not previously reported in Ranchod et al. [32]. Thus, our study leveraged the strengths of PLS analysis to examine age-related brain activity differences across two distinct load conditions (i.e., single vs. dual N-back tasks, [32]).

## Methods

This manuscript uses previously reported data from Ranchod et al. [32], mainly HbO data from young and older adults, adding HbR to the new PLS analysis. We report participants, their inclusion criteria, general procedure, and fNIRS layout and preprocessing. The full description of the task can be found in Ranchod et al. [32] and a condensed version of this task can be found in the Cognitive task and fNIRS acquisition section below.

### Participants

We recruited a total of 66 participants, 29 young adults (YA) and 37 older adults (OA) (June 1^st^, 2022-November 1^st^, 2022). Two participants terminated the experiment early, two older adult participants had a low Montreal Cognitive Assessment (MOCA) score of two standard deviations (SD) below the mean (*M)* (MOCA score *M* = 26.9; SD = 1.8; normal score range corresponds to 26–30), and 4 participants did not pass the fNIRS signal quality checks. Specifically, we calculated a structured noise index (SNI) to identify noisy channels (SNI < 2) and a group leverage analysis using the Brain AnalyzIR toolbox [53], described fully in Ranchod et al. [32] and similar to Meidenbauer et al. [40] and Zhuang et al. [56]. Although no participants were eliminated due to SNI values, two younger and two older adult participants had a subject leverage of p < .05 and were therefore removed from any further analyses. Thus, a total of 6 participants were removed from all the behavioural and PLS analyses. Our final sample consisted of 58 participants, 27 YA (18–27 years (yrs), *M* = 20.56, *SD* = 2.4 yrs, 18 Females; 9 Males) and 31 OA (64–84 yrs, *M* = 71.13, *SD* = 5.5 yrs, 21 Females, 10 Males, final MOCA score *M* = 27.1; SD = 1.6). All participants had normal or corrected-to-normal vision, at least 6 years of formal education (YA *M* = 15.07, *SD* = 1.33 yrs; OA *M* = 15.44, *SD* = 3.39 yrs), did not have any known neurological disorders, were not taking attention enhancing or psychoactive drugs, and were right-handed (self-reported). Participants attended one session lasting approximately 90 mins and were given either a 2% bonus credit or a $10 gift card for their participation. Participants were recruited from the local community and/or from the university and all gave written informed consent prior to any experimental procedure which was witnessed and signed by the investigators. The study was approved by the Thompson Rivers University research ethics committee.

### Procedure

#### fNIRS set up

Participants bilateral prefrontal brain activity was measured using fNIRS (Brite, Artinis Medical Systems, The Netherlands) while they completed 2 tasks with increasing cognitive load: a single and a dual 2-back tasks, reported and fully described in Ranchod et al. [32].

**Fig 1:**
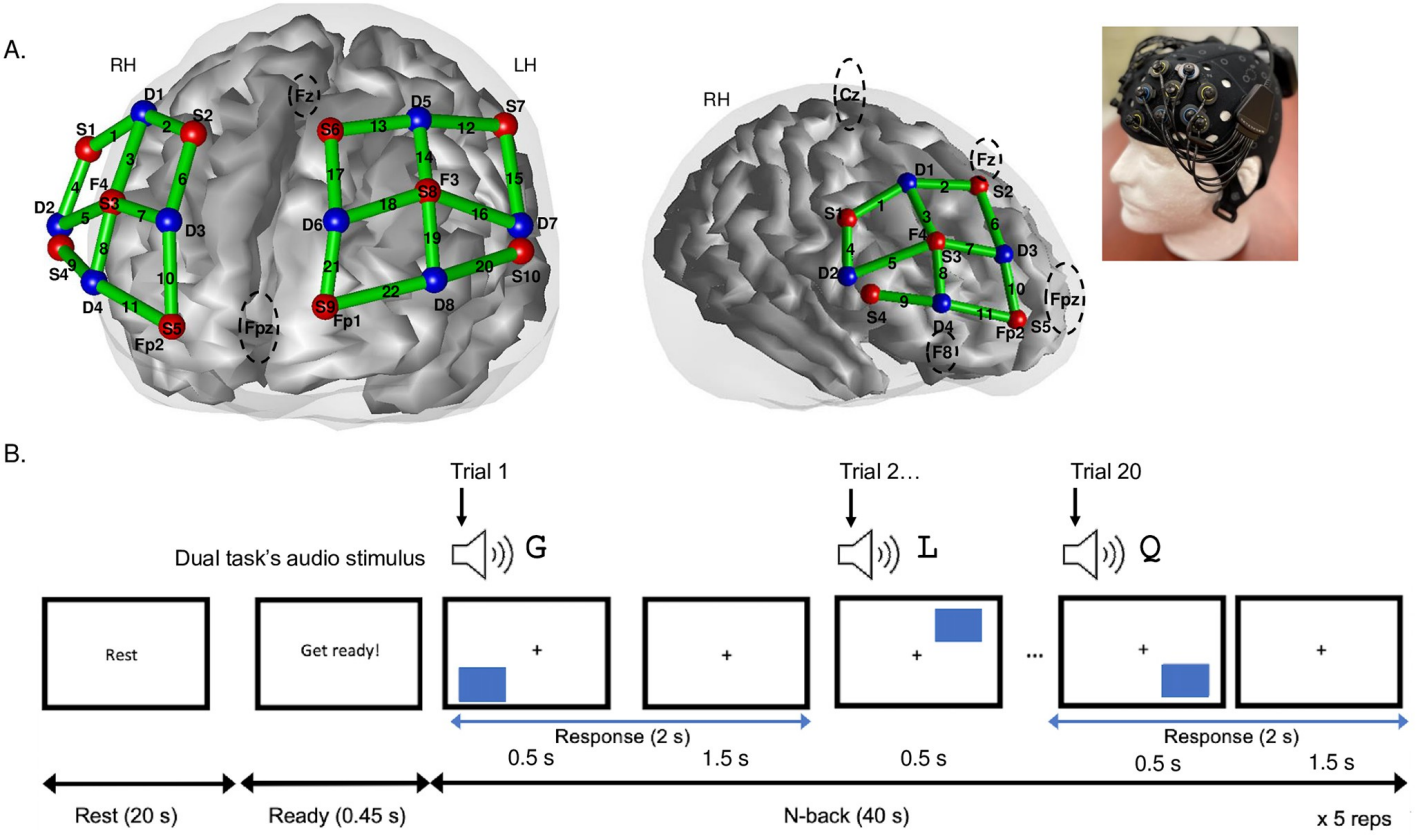
Optode montage and example of the dual 2-back condition. Upper panel A demonstrates de 2 x 12 array on right and left hemispheres (LH and RH, respectively) over the PFC; 2 channels are short separation channels (SSCH) used in the subject-level analysis’ GLM, thus, the diagram only identifies the 22 long channels for data collection. Panel B indicates that each visuospatial stimulus was also accompanied by an audio stimulus in the Dual task, whereas in the Single task, only a visuospatial stimulus was presented in each trial. A 20 s rest was followed by 40 s of a single or dual 2-back, repeated 5 times in total for both task-load conditions. A “D” key response for different from 2 trials back or “S” key response for same as 2 trials back was required in every trial. Adapted from Ranchod et al. [32].

The continuous wave fNIRS system comprised of 10 sources, emitting light (LED) at wavelengths of 650–950 nm, and 8 detectors placed on individual and adjustable optode holders on a neoprene headcap (Artinis, Medical Systems, The Netherlands) which was fitted on the participant’s head. Prior to fitting the headcap, we measured the participant’s head to make sure that the headcap was the correct size and marked the vertex or Cz on the participant’s head, according to the 10–20 International System [57]. The pre-marked vertex or Cz on the headcap was then aligned with each participant’s marked Cz. Furthermore, the locations of sources and detectors were also digitized in reference to the vertex, inion, nasion, and preauricular landmarks using a digitizing system (Patriot, Polhemus). The exported locations of sources, detectors, and source-detector pairing (i.e., channels) were registered to a 3D brain template (Colin27 atlas) in the Brain AnalyZIR toolbox [53] (Fig 1, panel A).

The source-detector pairings made up a 2 x 12 array resulting in a total of 24 channels (CH) over bilateral prefrontal cortices (12 on the left and 12 on the right hemispheres) (see Fig 1, panel A). From those 24 channels, two were short separation channels (SSCH, 1.5 cm) used to eliminate unwanted physiological noise (e.g., scalp blood flow) [58], whilst the rest (22 CH) were long separation channels at an optimal 3 cm apart to measure cortical differences in oxy (HbO) and deoxyhemoglobin (HbR) concentrations. Prior to data collection we made sure that the fNIRS signals were optimal by clearing the hair under all optodes, using distance safeguards to ensure that optode pairing distance remained optimal (at 3 cm), and tested for ambient light interference.

Our array placement was based on previous findings from N-back tasks using fMRI [24] and fNIRS [40, 47]. Additionally and in accordance with previous reports, our region of interest analysis (ROI) [32], which used polhemus digitized locations and implemented a depth map function [53] to estimate the distance between a channel and the surface of the cortex, identified the antero-medial frontal cortex (BA 10), the dorsolateral prefrontal cortex (DLPFC) (BA 9 and BA 46), the inferior frontal gyrus (IFG) (BA 44 and BA 45), and the superior frontal gyrus (BA 8) as 6 Tailarach daemon regions of the Colin27 atlas covered by our array in both hemispheres.

#### Cognitive task and fNIRS data acquisition

All tasks and stimuli were designed using E-prime 3.0 (Psychology Software Tools Inc., PA, USA) and presented on a computer laptop (Dell Latitude 3410, 14” HD, 1920 x 1200 resolution) positioned in front of the seated participant. For the 2-back single task, a visuospatial stimulus was used, which corresponded to a blue rectangle appearing in sequence across 6 possible locations, whilst for the dual task, the visuospatial and auditory stimuli were presented simultaneously (Fig 1, panel B). The dual task was therefore a high load task requiring additional cognitive resources [54, 59]. The auditory stimuli consisted of audible letters (i.e., G, H, K, L, P, or Q) presented alongside the blue rectangle via the laptop’s speakers. Participants were required to press either the “S” key or the “D” key depending on whether the current rectangle was in the same or different location as 2 trials back, respectively, for the single task. In the dual task, participants had to respond using the same “S” or “D” buttons but had to determine whether the visual and/or auditory stimuli were repeated or different from two trials back. All stimuli were presented for 500 ms and participants had a total of 2000 ms to respond [32]. We used a 2-back as this task has been shown to be challenging but not too demanding compared with an easy 1-back, which we argue could inflate differences between age-groups [23].

For fNIRS design, 20 s rest periods preceded 40 s of continuous single or dual 2-back trials in which behavioural responses (i.e., reaction times and accuracy) were collected and exported from Eprime. Participants performed 20 s of rest and 40 s of 2-back blocks 5 times for the single and 5 times for the dual task load conditions (also see Fig 1B). Each load condition had a ratio of 20% of target trials (i.e., trials requiring a “S” key response) and 80% of non-target trials (i.e., trials requiring a “D” key response). Participants then had to press a key in every trial (i.e., “S” or “D”) to control for motor responses in the fNIRS signals. All participants received detailed instructions and practice with feedback prior to each 2-back task and were asked to be as fast and accurate as possible for all stimuli presented.

All fNIRS data was collected using Oxysoft (Artinis, Medical Systems, The Netherlands, version 3.2.51.4) and sampled at 25 Hz. Distributed component object model (DCOM) methods were used to insert events (“N-back”) from Eprime into Oxysoft. Participants completed the single followed by the dual task [59] and received practice (with feedback) prior to each task.

### fNIRS data processing for PLS analyses

As described previously in Ranchod et al. [32], all fNIRS data obtained using Oxysoft was converted to SNIRF files and imported in MATLAB (version 9.13.0, 2022b, Mathworks Inc., Massachusetts) and processed using Brain AnalyzIR toolkit [53]. Raw data was converted to optical density and to HbO and HbR concentrations (μM) using the modified Beer-Lambert law [60].

Quality control of the datasets was conducted pre subject level analyses to remove participants who had poor quality HbO and HbR signals on multiple channels. To determine whether a channel was of poor quality the structured noise index (SNI) was calculated according to Zhuang et al. [56]. The SNI examines the channel data for high systematic noise with higher values indicating greater magnitudes of noise of a repeating nature. For instance, a signal with a high SNI will contain periodicity due to arterial blood pressure oscillations. As in Ranchod et al. [32] we identified poor signals as those with an SNI < 2. As with our previous work, the number of “bad” channels was only 0.4% of HbO and 1.1% of HbR channels and as such, no participant data was removed at this stage.

We implemented a subject-level general linear model (GLM) in Brain AnalyzIR toolbox to quantify task related hemodynamic responses as beta coefficients based on the canonical hemodynamic response function (HRF), as suggested by Santosa et al. [53]. To obtain these beta coefficients for subsequent PLS analyses we implemented the GLM with short-separation channels as regressors and used an autoregressive iteratively reweighted least-squares model (AR-IRLS). This model uses pre-whitening and robust regression to remove motion and auto-correlated noise from the fNIRS signal [61]. This method is well described by Huppert [52].

After obtaining the subject-level statistics, we implemented a second quality control routine to remove participants who data would disproportionately affect PLS analyses. To do this we completed group leverage analyses and identified 2 YA and 2 OA participants whose leverage was statistically significant at p > .05. These participants beta coefficients and behavioral data were removed prior to PLS analyses.

## Analysis

### Behavioural analysis

Behavioural data analysis is described in Ranchod et al. [32], which included the extraction of reaction times (RTs) of correct trials and accuracy in error rates. Average values for these variables across trials within single and dual tasks were calculated for each participant within each group. These values of performance were used in the PLS analyses. In total, there were twenty-seven YA and thirty-one OA participants each with single and dual values for RTs and error rates.

### Behavioural PLS analyses

Behavioural PLS analysis was implemented similar to Meidenbauer et al. [40] to determine relationships between behaviour characteristics and brain oxygenation (HbO) and deoxygenation (HbR) patterns measured using fNIRS across groups and cognitive load levels of the N-back task. This is a multivariate, data-driven approach used to examine these brain-behaviour relationships as previously described by Krishnan et al. [62]. We were interested in examining the relationship between behaviour and brain HbO and HbR depending on the task difficulty (single vs. dual) in our two populations (young and older adults). Beta-coefficients from the subject-level GLM were exported from Brain AnalyzIR toolkit after processing and removal of unsuitable participants as outlined above. For each behavioural PLS analysis (behaviour x HbO or HbR), each participant (YA n = 27, OA n = 31) had 44 brain activity values (single and dual N-back task each with 22 channels) and two behavioural values, i.e., RT and error rates.

The behavioural PLS was performed using the MATLAB toolkit developed by McIntosh and Lobaugh [43] available at Baycrest Rothman Research Institute (https://www.rotman-baycrest.on.ca/index.php?section=84). The command-line PLS scripts enable relationships between the behaviour and brain activity values to be assessed in MATLAB. Behavioural PLS involves single value decomposition (SVD) of a covariance matrix. In the case of this experiment matrix X is the brain beta coefficients and matrix Y is the behavioural variable (i.e. error rate). These matrices are first mean centred and normalized within the single and dual N-back tasks. The brain beta coefficient matrix was 58 participants x 22 channels, but this was stacked so that the 27 YA participants were in the first rows followed by all 31 OA participants, and there were 2 of these matrices corresponding to single and dual tasks. The behavioural matrix Y was a vector 58 x 1 of YA stacked above OA participant data and there were two of these matrices containing the single and dual N-back task data. The stacking order and number of participants included in each group is provided as inputs to enable group specific analyses across conditions. Since there were two behavioral variables assessed (RT and error rates) and two hemodynamic responses (HbO and HbR) a total of 4 behavioural PLS analyses were conducted.

The product of matrices X and Y was calculated (e.g. HbO’ * RT), which yielded a vector of correlations of size 1 x 22 and there were four of these vectors (2 groups x 2 N-back levels). The resultant 4 x 22 cross-product matrix R was the input for subsequent SVD which creates R = UΔV^T^, where the singular vector U (behaviour saliences) is the decomposition of R in behaviour/condition/group space, the singular vector V (brain saliences) is the decomposition of R in brain oxygenation or deoxygenation space, and Δ is the diagonal matrix of singular values that gives the weighting of each of the singular vectors. Thus, U represents the differences in the brain-behaviour correlations between the two levels of the N-back task across the two groups, whilst the V represents the fNIRS channel-dependent differences in this brain-behaviour correlation by N-back task across the two groups.

The goal of these PLS analyses was to identify the linear combination of condition and brain activity that maximized their covariance amongst the young adult and older adult groups. These weighted patterns are determined from the behaviour (U) and brain (V) saliences described above and are referred to as latent variables (LVs). The LVs (4 in these models) maximize the covariance between U and V as a function of N-back task across groups. To obtain these LVs, 10,000 permutation tests were performed to obtain the p-values for these 4 LVs per model (e.g. HbO and RT across 2 N-back levels in the YA and OA groups). To obtain 95% confidence intervals for the mean correlation between fNIRS activity and performance (e.g. RT) across N-back task and across groups 10,0000 bootstrapped samples with replacement were performed. Bootstrap ratios (BSR) (salience[weight]/SE[reliability]) provide an estimate of the reliability of brain-behaviour relationships within each channel and when above a specific threshold (|2| in this analysis) are considered important. Also, the larger the BSR the stronger and more consistent the contribution of that particular fNIRS channel activity to the LV. As explained by Meidenbauer et al. [40], these BSR can be interpreted as z-scores.

## Results

For detailed behavioral results, please see Ranchod et al. [32]. In summary, in Ranchod et al. [32] we reported that both age-groups demonstrated slower RTs and higher error rates in the dual vs. the single task (all *p* < 0.05); thus, the dual task increased cognitive load across the age-groups. In addition, although OA’s demonstrated slower RTs compared with YA when responding to targets (i.e., trials with “S” responses), and were particularly slower in the dual task (given by a 3-way interaction, F(1, 56) = 5. 527, *p* = 0.022, η2 = 0.001), they did not show significant differences in error rates compared with YA [32]. Although unexpected, similar error rates provided an opportunity to assess age-related differences in brain activity without the confound of distinct levels of performance. Furthermore, our previous univariate fNIRS analyses of HbO channel and ROI activity revealed that YA had more active channels in the single vs. dual task, whilst OA demonstrated an increase in brain activation (i.e., more channels, higher beta values) from the single to the dual (all *p* < 0.05). OA also demonstrated more bilateral PFC activation vs. YA across both task complexities (see Ranchod et al., [32]).

### PLS analyses

For all PLS analyses involving brain oxygenation, deoxygenation and behaviour measures, four LVs were assessed, and in each case, one LV was significant. All other LVs were not significant (*p* > 0.9). Note that increases in oxygenated hemoglobin and decreases in deoxygenated hemoglobin (i.e., larger reductions or more negative values of deoxygenated hemoglobin) would correspond to increases in brain activity; whilst decreases in HbO and increases in HbR (i.e., less negative HbR values) corresponded to decreases in brain activity [63–65]. Thus, for example, both positive relationships between HbO and performance measures, and negative correlations between HbR and performance measures would correspond to increases in brain activity across performance; and both, negative relationships between HbO with performance measures, and a positive relationship between HbR and performance would indicate a reduction in brain activity. For ease, we use greater or more brain activity (i.e., increases in HbO and decreases of HbR) and decreased or reduced brain activity (i.e., decreases in HbO and increases of HbR) to describe the results. Scatterplots of all relationships across channels, conditions and groups are depicted in Supplemental Information (S1–S4 Figs).

#### Reaction time and oxyhemoglobin (HbO)

**Fig 2:**
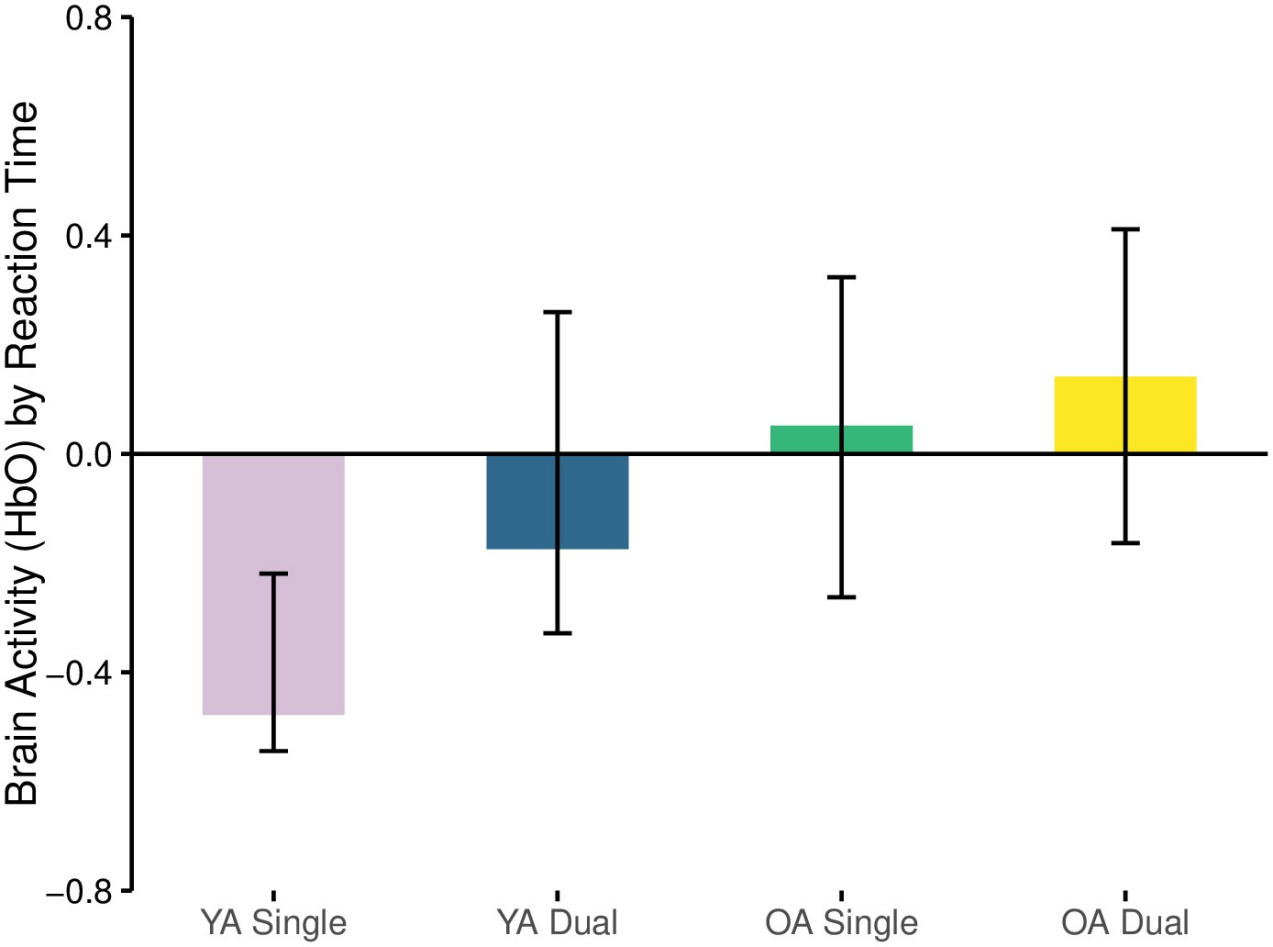
Correlation between brain activity (HbO) and RT separated by age-group and task load condition.

The first LV relating oxyhemoglobin and reaction time was significant (*p* = 0.04) and explained 63.9% of the crossblock variance. Amongst the 4 relationships assessed (YA and OA in Single and Dual task conditions) reaction time and oxyhemoglobin concentration during the single N-back task in YA was negative (r = -0.478, Fig 2). There were no relationships between oxyhemoglobin and reaction time in the OA group across cognitive load (Fig 2, Table 1).

**Table 1:** Summary of PLS results for HbO and HbR vs. RT and Error rate.

LV	*p* value	Crossblock variance (%)	Group	Condition	Relationship^a^ (r)	Channels
HbO vs. RT	0.04	63.9%	YA	Single	r = -0.478	5, 9,19
				Dual	-	
			OA	Single	-	
				Dual	-	
HbR vs. RT	0.055*	44.3%	YA	Single	r = 0.49	5, 9,11, 22
				Dual	-	
			OA	Single	r = -0.346	
				Dual	r = -0.335	
HbO vs. Error rate	0.014	70.8%	YA	Single	r = -0.421	1, 3, 5, 6,12, 14, 15, 18, 22
				Dual	-	
			OA	Single	-	1, 5, 6, 14, 18, 22
				Dual	r = -0.275	
HbR vs. Error rate	0.01	57.5%	YA	Single	r = 0.357	3, 4, 5, 6, 7, 8
				Dual	-	
			OA	Single	r = -0.364	
				Dual	-	

(*) Demonstrates a trend and (-) demonstrates a null relationship.

^a^ A negative relationship (r) in HbO indicates increased brain activity with decreased RT or error rate; whilst a positive relationship indicates greater brain activity with increased RT or error rates. A positive relationship in HbR indicates increases in brain activity as RT or error rates decrease; whilst the negative relationship in HbR demonstrates that as RT or error rate increases there is more brain activity or decreases in deoxyhemoglobin.

**Fig 3:**
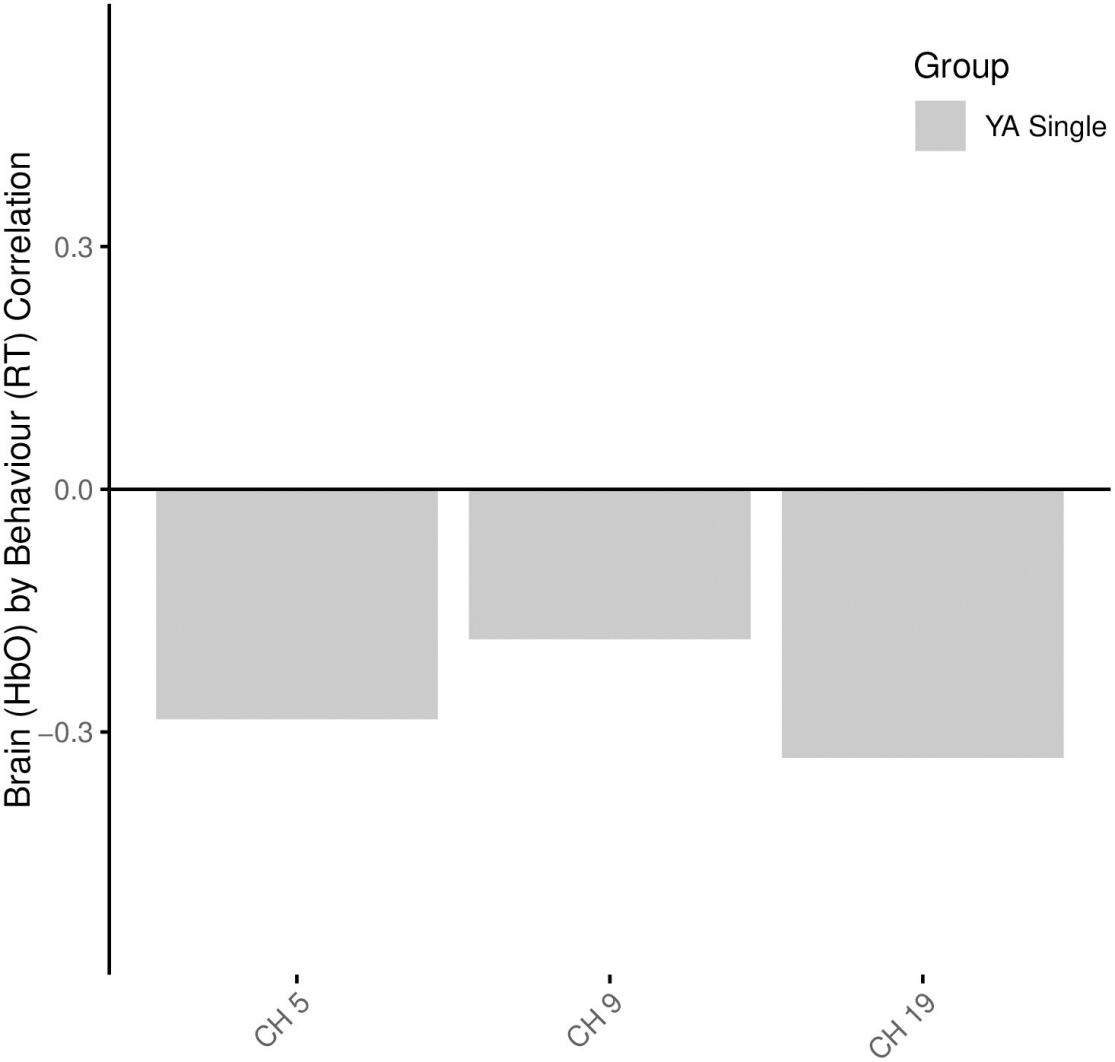
Brain activity (HbO) and RT correlation across relevant channels (BSR > 2). Only YA single task’s ch 5, 9, and 19 are shown.

**Fig 4:**
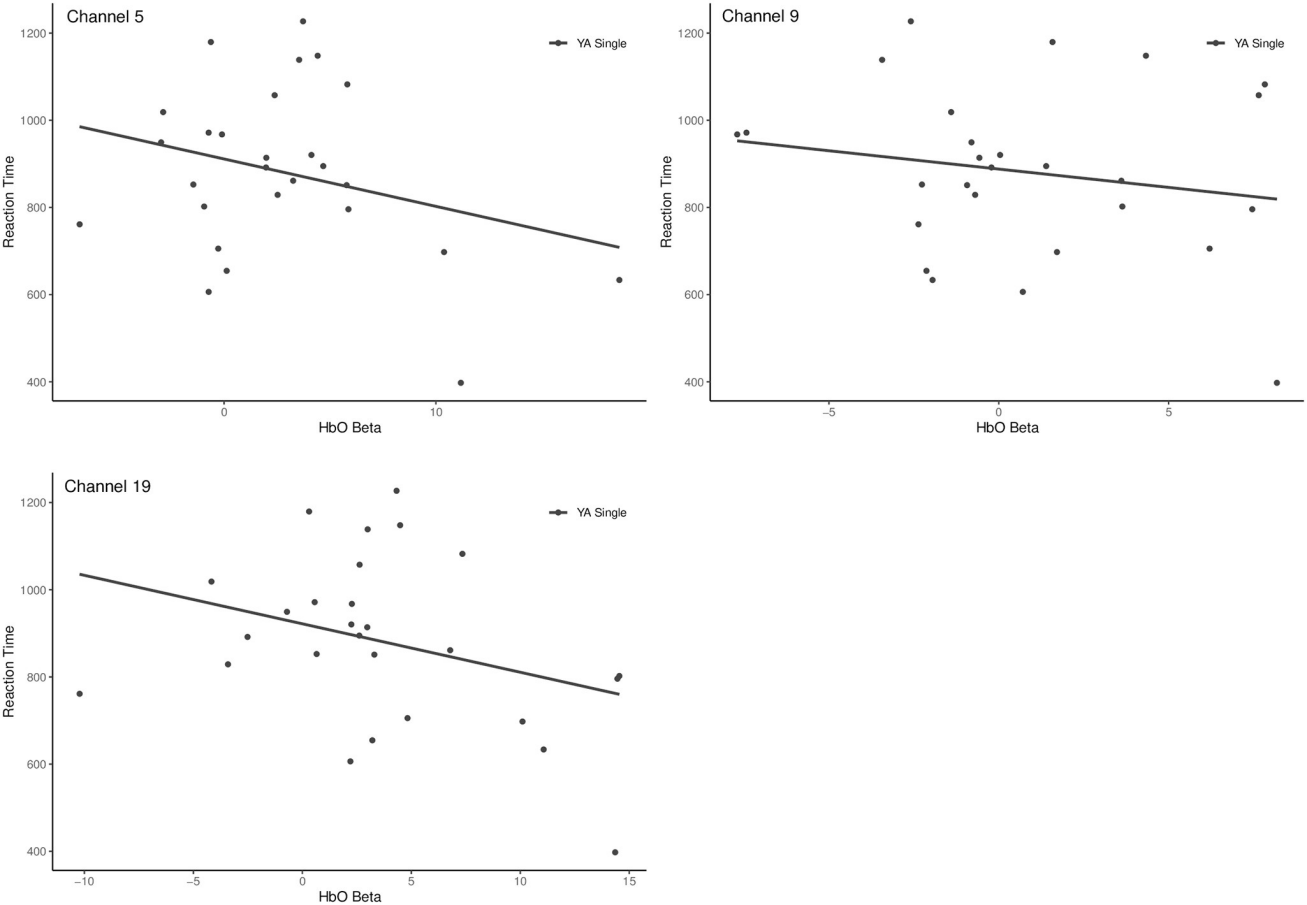
Scatterplots of the correlation between HbO betas and reaction time across the areas of the DLPFC in the YA group during the single task. Only channels with BSR > 2 are displayed.

The negative relationship suggests that oxyhemoglobin concentration and brain activity was reduced as reaction times increased amongst the YA during the single task. Areas of the brain that were reliably involved in this directional pattern (BSR > 2) were in the dorsolateral prefrontal cortices (DLPFC, BA46) across both hemispheres (channels 5, 9, in RH and channel 19 in LH) (Fig 3). Select scatterplots of specific channels are depicted in Fig 4.

#### Reaction time and deoxyhemoglobin

**Fig 5:**
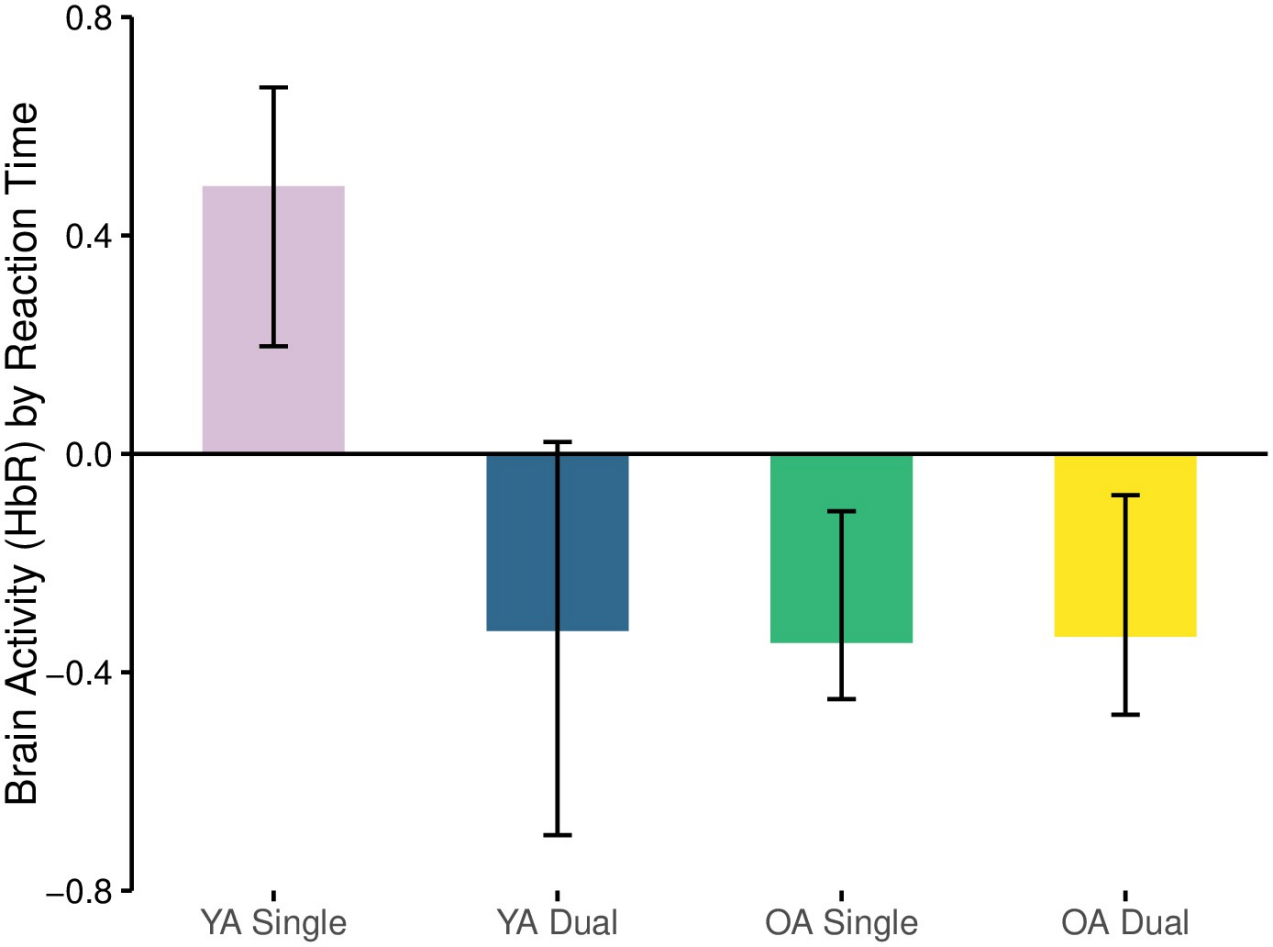
Correlation between brain activity (HbR) and RT separated by age-group and task load condition.

The first LV relating reaction time and deoxyhemoglobin concentration was approaching significance (*p* = 0.055) and explained 44.3% of the crossblock variance. This relationship was positive amongst YA when performing the single task (r = 0.49), yet negative amongst OAs in the single task (r = -0.346) and the dual task (r = -0.335) (Fig 5, Table 1). This suggests that reaction time increased in concert with decreases in brain activity in YA’s, demonstrated by increases in deoxyhemoglobin, whereas when reaction time increased in OAs there was also an increase in brain activity, demonstrated by a reduced deoxygenation. No other relationships were apparent.

**Fig 6:**
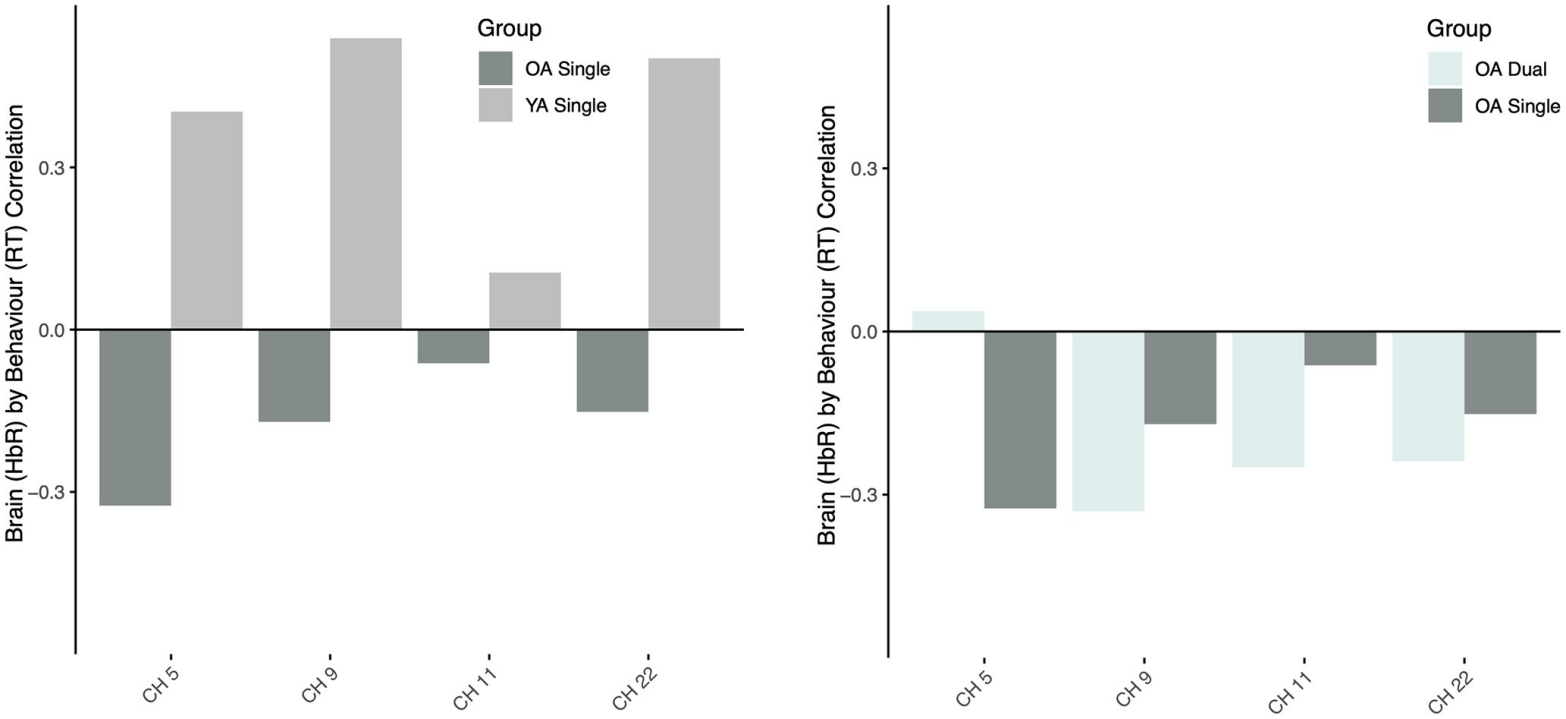
Brain activity (HbR) and RT correlation across relevant channels (BSR > 2). Results show relevant channels in the single task between OA and YA (left) and OA’s single vs. dual task (right).

**Fig 7:**
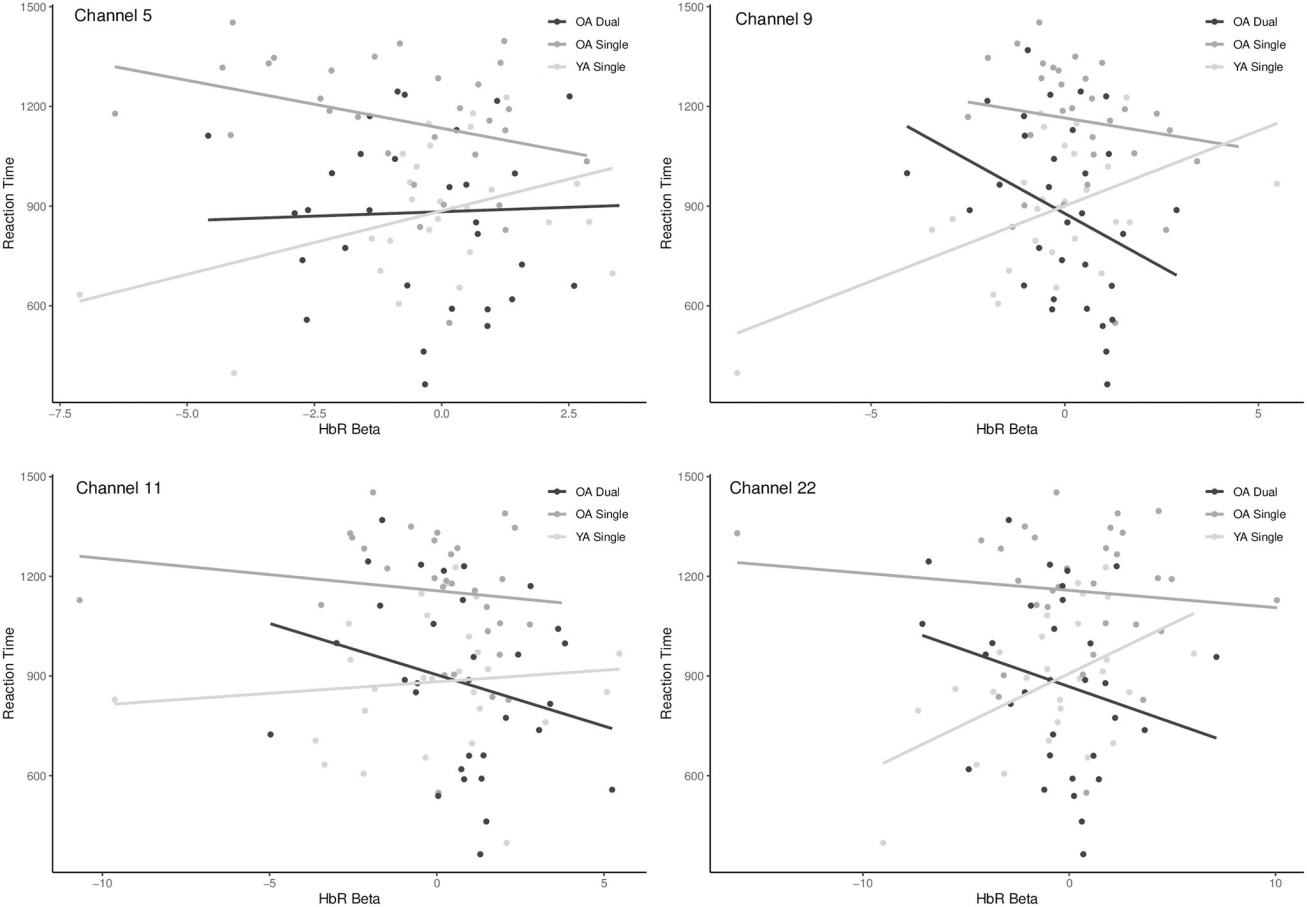
Scatterplots of the correlation between HbR betas and reaction time across the areas of the DLPFC in the YA group during the single task and both single and dual task in OA. Only channels with BSR > 2 are displayed.

Areas of the brain that reliably contributed to these opposing relationships across age-groups were the right dorsolateral prefrontal cortex (BA46), and the anterior prefrontal cortex (BA10) with more of a relationship within the right hemisphere (channels 5, 9, 11 in RH, and channel 22 in LH) (Fig 6). In OAs, the direction (i.e., negative) was the same across most channels, except channel 5, for single and dual load conditions (Fig 6, Table 1). Scatterplots of specific channels of are depicted in Fig 7.

#### Error rate and oxyhemoglobin

**Fig 8:**
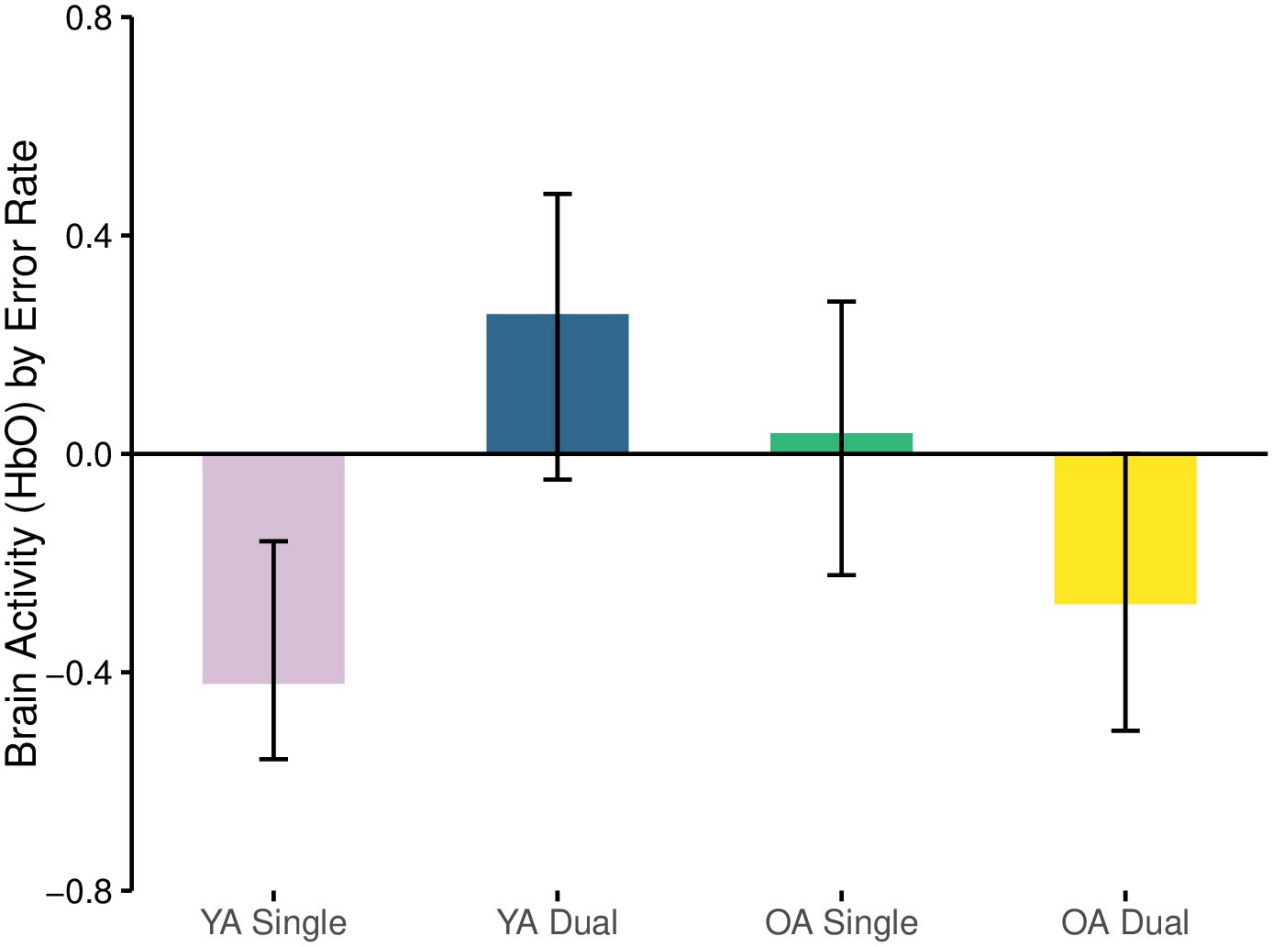
Correlation between brain activity (HbO) and error rate by age-group and task load condition.

The first LV relating error rate and oxyhemoglobin concentration was significant (*p* = 0.014) and explained 70.8% of the crossblock variance. This relationship was negative for both YAs when they performed the single task (r = -0.421) and OAs when they completed the dual task (r = -0.275) (Fig 8, Table 1). No other relationships were apparent.

**Fig 9:**
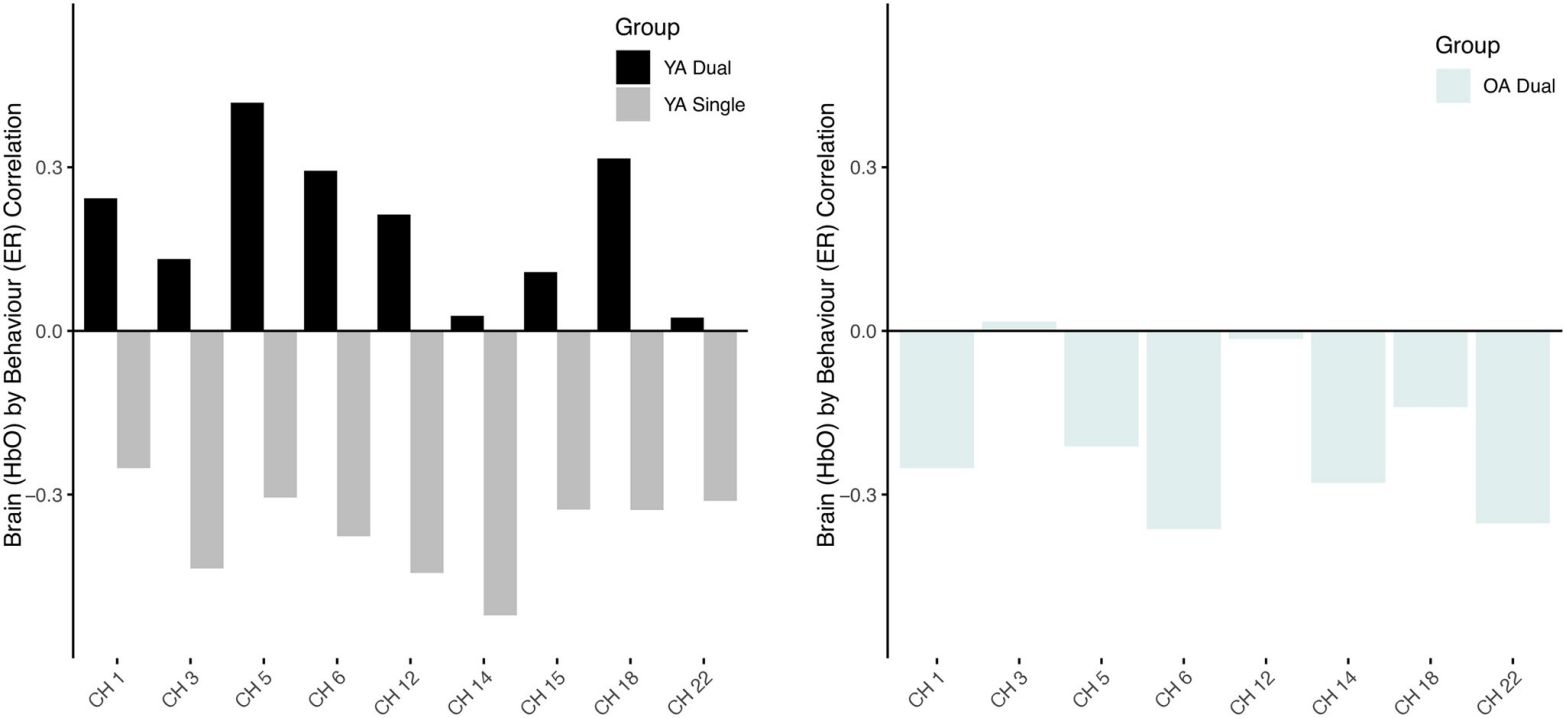
Brain activity (HbO) and error rate (ER) correlation across relevant channels (BSR > 2). Results show relevant channels in the single task and dual task in YA (left); and relevant channels with mostly, a negative relationship between HbO and perforamnce in OA dual task.

**Fig 10:**
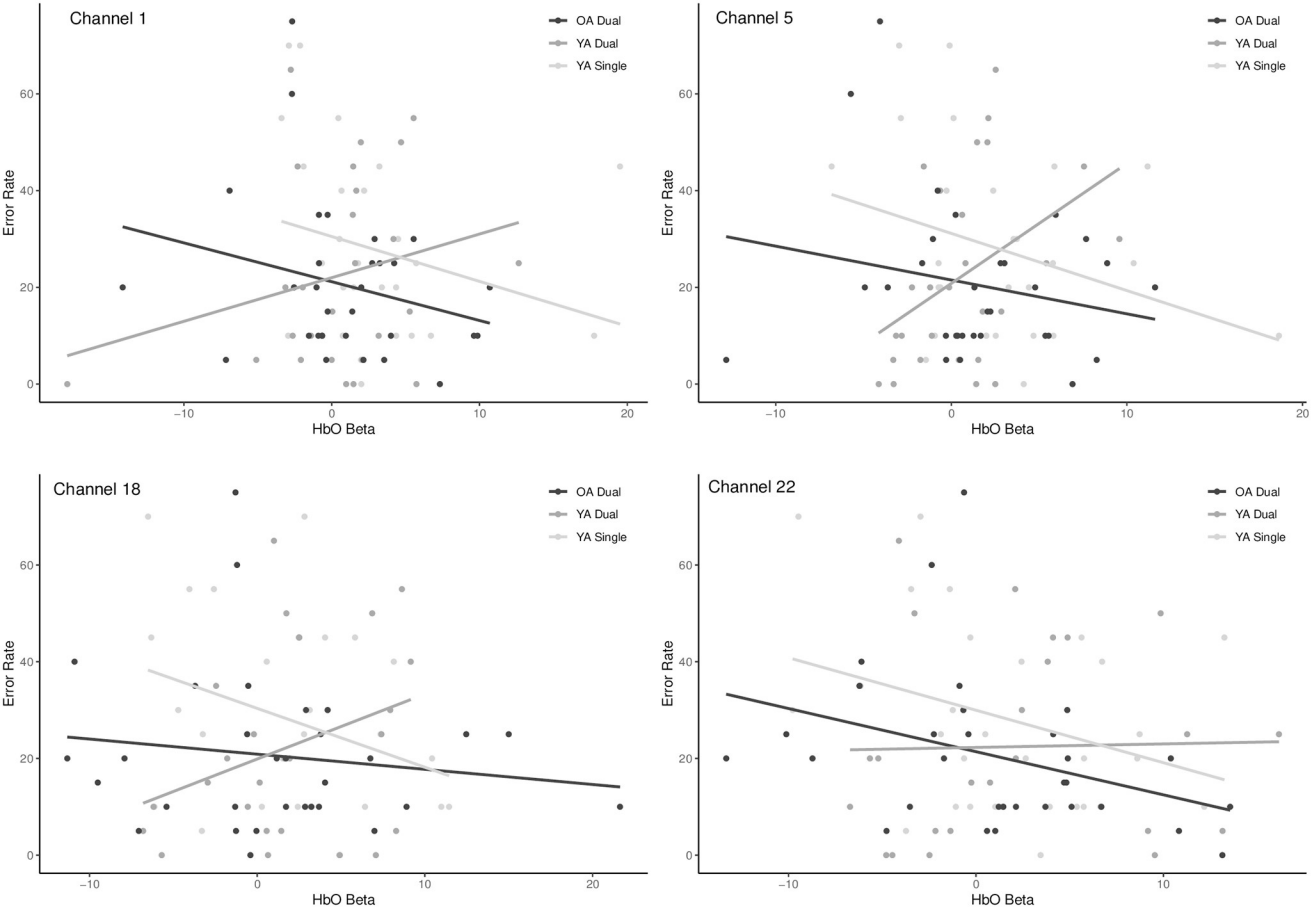
Scatterplots of the correlation between HbO betas and error rates across select areas of the frontal eye field, DLPFC, anterio-medial and anterior prefrontal cortices (Channels 1, 5, 18 and 22) in the both single and dual task in YA and only the dual task in OA. Only channels with BSR > 2 are displayed.

The negative relationships suggest that as error rate increased brain activity decreased (i.e., HbO decreased). This relationship was most reliable (BSR > 2) across the same regions as the reaction time and oxyhemoglobin relationship, meaning it involved both hemispheres and specifically the frontal eye fields (BA8) antero-medial frontal (BA10), dorsolateral prefrontal (BA46) and medial prefrontal cortices (BA9) (channels 1, 3, 5–6, 12, 14–15, 18 and 22) (Fig 9, Table 1). Of note, the OAs had fewer areas of the brain involved in this relationship (channels 1, 5–6, 14,18, 22). Scatterplots of specific channels are depicted in Fig 10.

#### Error rate and deoxyhemoglobin

**Fig 11:**
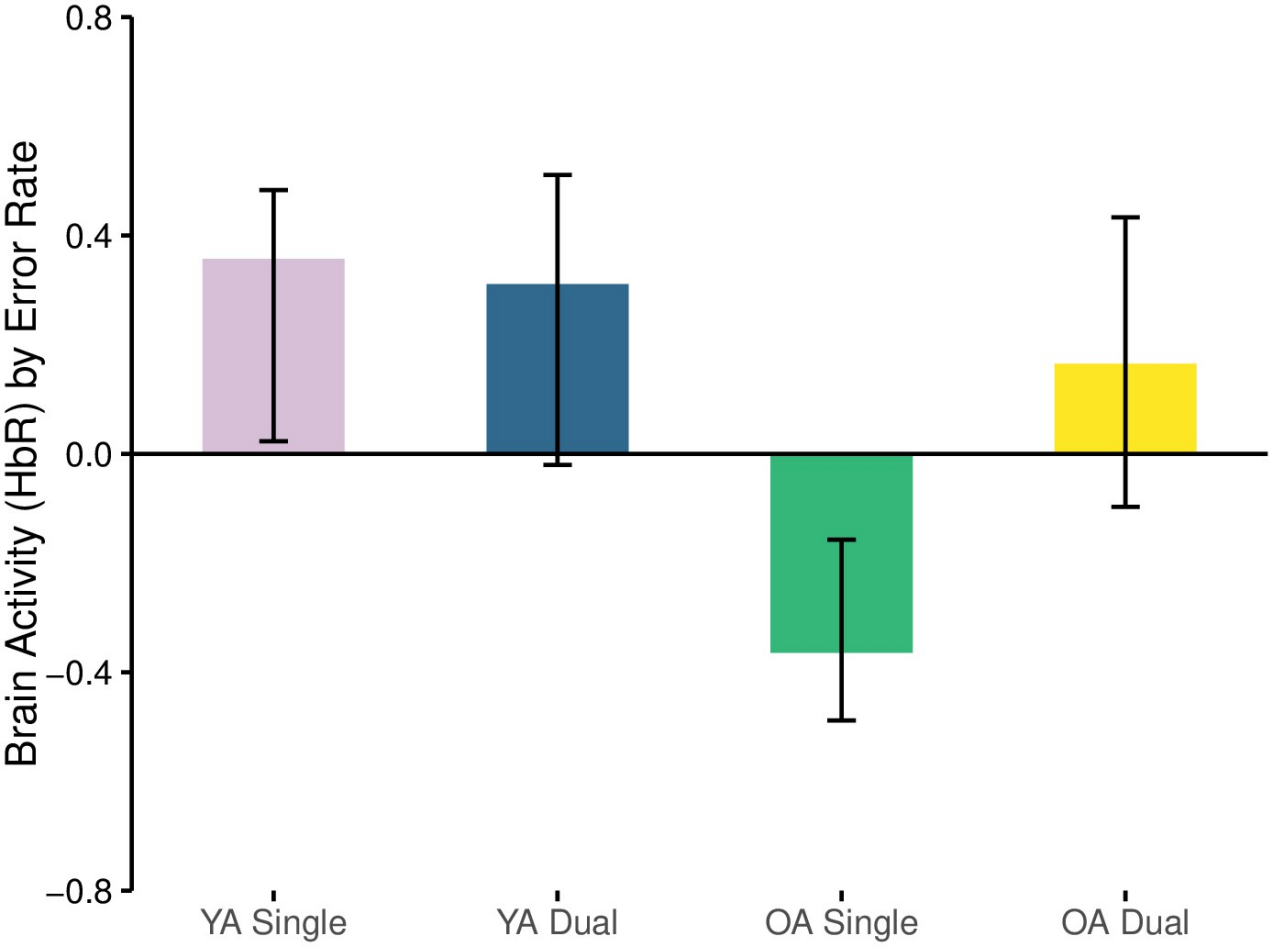
Correlation between brain activity (HbR) and error rate separated by age-group and task load condition.

The first LV relating error rate and deoxyhemoglobin concentration was significant (*p* = 0.01) and explained 57.5% of the crossblock variance. The relationship was negative amongst OAs when they completed the single task (r = -0.364) and different from all other relationships which were all positive (Fig 11, Table 1). Of the positive relationships, YAs had similar responses when performing both the single and dual N-back tasks but only the single task was likely important and reliable (r = 0.357) (Fig 11).

The negative relationship in the OAs suggests that as error rate increased, brain activity also increased as shown by a decrease in the concentration of deoxygenated haemoglobin. Whereas amongst the YAs, the positive relationship suggests that during the single task they had greater error rates in concert with decreases in brain activity (i.e., deoxygenation was also highest) (Fig 11).

**Fig 12:**
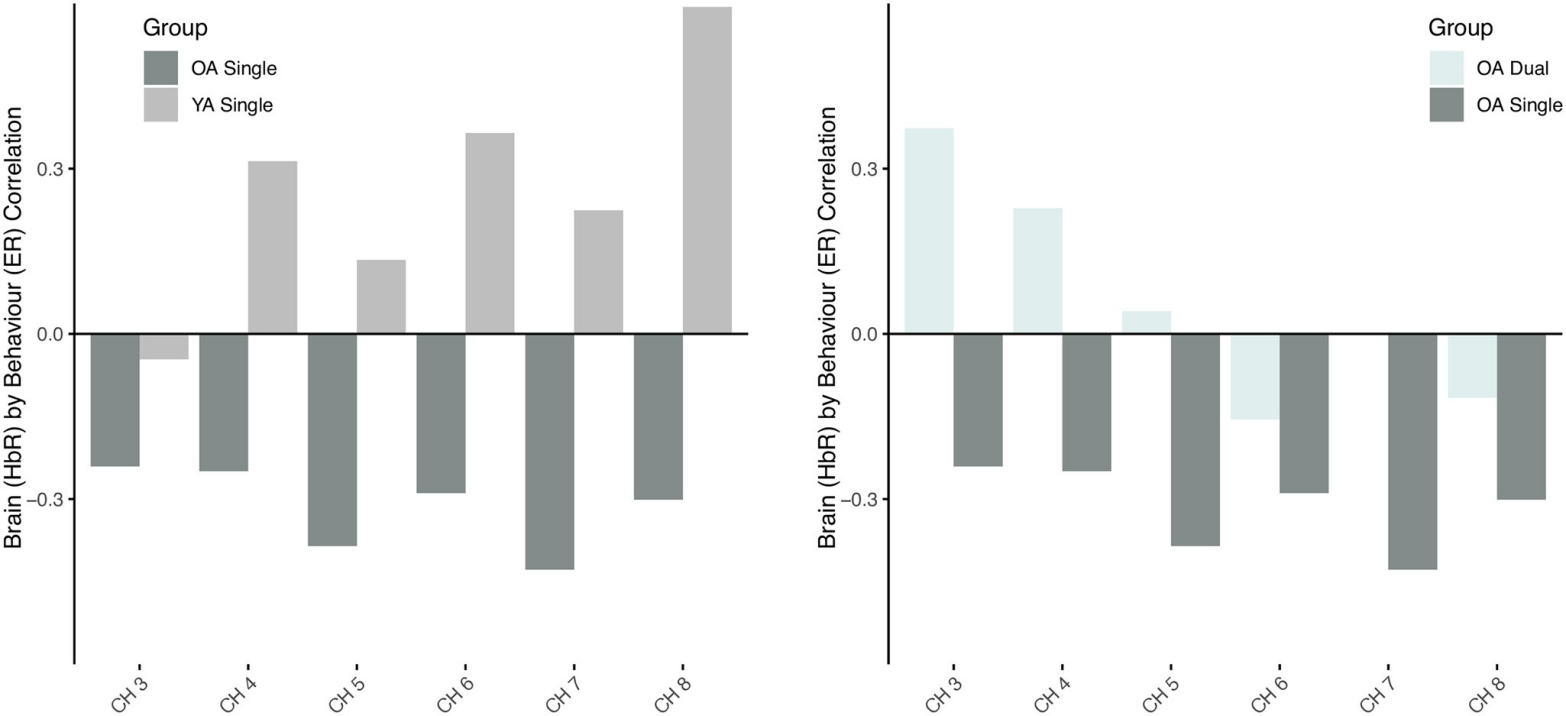
Brain activity (HbR) and error rate (ER) correlation across relevant channels (BSR > 2). OA and YA single task was significant but with contrasting relationships in most channels (left). Results also demonstrate the negative relationship between HbR and performance in the dual task (all channels) vs. the non significant single task (right).

**Fig 13:**
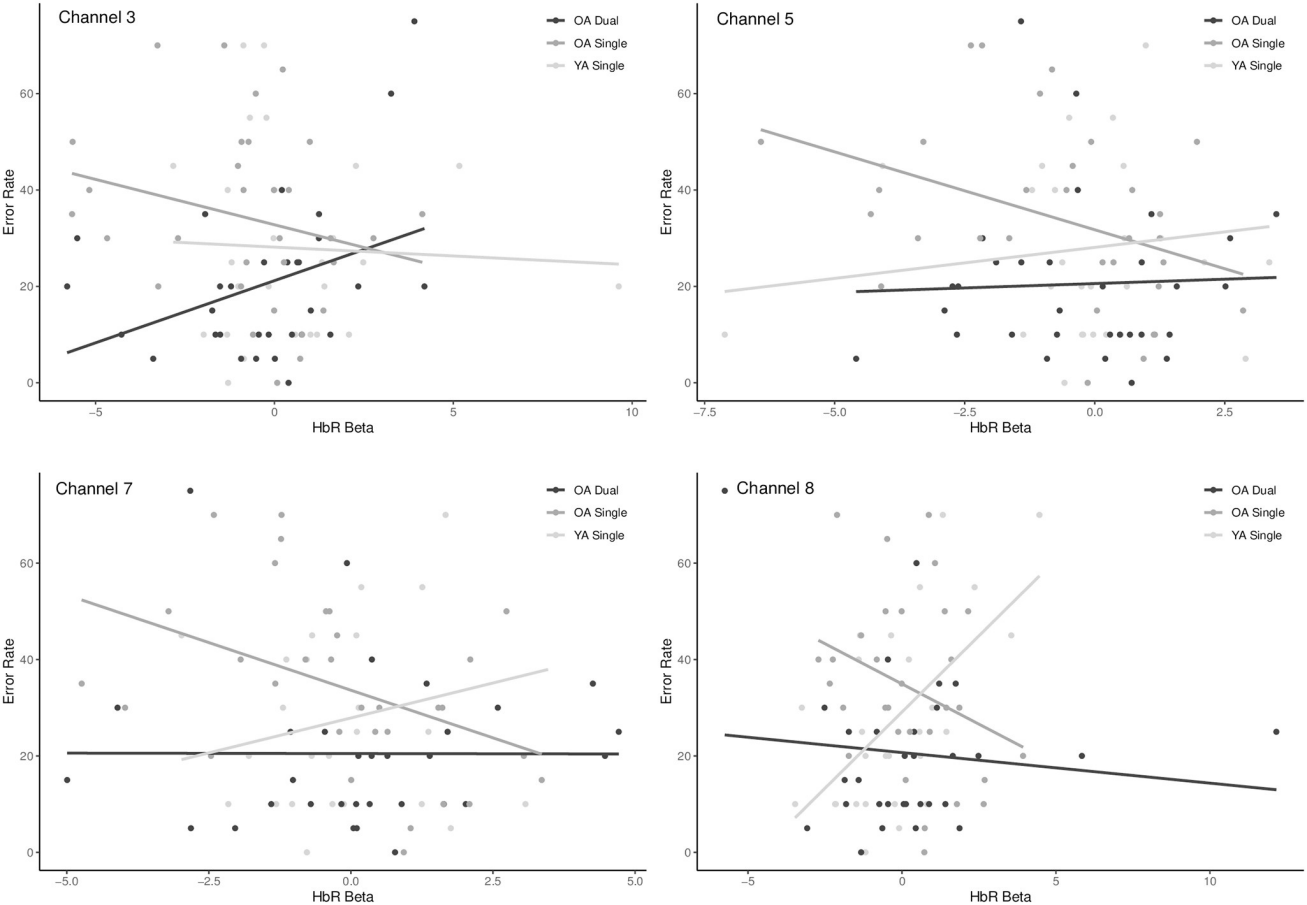
Scatterplots of the correlation between HbO betas and error rates across select areas of the right frontal eye field, DLPFC, medial and anterior prefrontal cortices (Channels 3, 5, 7 and 8) in the both single and dual task in OA and only the single task in YA. Only channels with BSR > 2 are displayed.

Areas of the brain that reliably contributed to these opposing relationships in the different populations were limited to the right hemisphere and included the frontal eye fields (BA8) antero-medial frontal (BA10), dorsolateral prefrontal (BA46) and medial prefrontal cortices (BA9) or channels 3–8 (Fig 12, Table 1). Scatterplots of specific channels are depicted in Fig 13.

## Discussion

Our previous channel and ROI analyses revealed differences in HbO activity across cognitive loads and between age-groups, with YA showing decreased brain activity in the more complex dual task relative to the single task and OA increasing brain activity (i.e., more active channels) in the dual relative to the single task [32]. These findings go against the notion that OA’s brain activity attenuates earlier at lower loads vs. YA (CRUNCH model [23, 26]) but are in line with other reports showing steady increases in brain activity in concert with increases in task complexity [25, 28, 29]. Behaviourally, error rates were higher in the dual task vs. the single task overall but did not differ between age-groups at any task load [32].

The present study’s behavioural PLS analysis allowed us to examine the relationship between performance (i.e., RT and error rate) and brain activity (i.e., HbO and HbR) and importantly, assess whether this relationship is influenced by cognitive load using multivariate approaches that directly correlated performance with brain activity across task loads. Determining such direct relationships is important since age-related neurocognitive patterns, like compensation, are identified and characterized by implementing tasks with distinct cognitive loads [23, 25–27, 66]. Notably, findings from reports implementing such protocols are mixed regarding OA’s brain activity patterns as task load increases (Jamadar [25] and Ranchod et al. [32] vs. Mattay et al. [23] and Bauer et al. [67]).

Our PLS analysis revealed a negative oxyhemoglobin significant relationship and a positive deoxyhemoglobin concentration trend with RT amongst YAs, demonstrating that as HbO increased and HbR decreased, as expected their RT got faster in the single task. Similarly, PLS analysis involving accuracy as the behavioural measure revealed a negative relationship between HbO and error rate, indicating that as HbO levels increased, and HbR levels decreased, error rates decreased in YA’s single task. These relationships between behavioral measures of performance and fNIRS brain activity (HbO and HbR) may indicate that higher levels of prefrontal neural engagement are associated with better performance (i.e., faster RTs and better accuracy) in YA, which may reflect more efficient allocation of cognitive resources or enhanced neural processing leading to improvements in performance. Furthermore, the multivariate approach uncovered many channels (9 channels in total, 4 on RH and 5 on LH, in HbO vs. error rate) involved in this single task in YA that were additional and not observed in our previous channel or ROI analysis in which behaviour measures were not (directly) accounted for. It is important to note that our single task was a 2-back working memory task, which differs from the low complexity task used previously [23, 40]. For example, Meidenbauer et al. [40] used 3 levels of an N-back task in their behavioural PLS analysis to examine brain-behaviour relationships in YA and found less brain activation (HbR related) associated with better performance in their 1-back task. They suggested this indicated increased automaticity and enhanced neural efficiency in this relatively simple, low load task. In the more complex tasks (i.e., 2-back and 3-back), such automaticity and corresponding brain activity attenuation was not observed; instead, extensive PFC recruitment was needed to meet the higher demands on attention and working memory [40]. Meidenbauer et al.’s [40] PLS analysis revealed no relationship between accuracy and brain activity in the 2-back but a positive relationship in their 3-back, demonstrating that decreased HbR (i.e., increased brain activity) was associated with better performance in the more demanding task compared with the simpler 1-back. This ability to enhance brain activity at higher loads demonstrates young adults’ enhanced neural capacity rather than neural efficiency [17]. Thus, for YA, our single 2-back was complex enough to elicit high metabolic demands which resulted in bilateral neural activation to meet the task requirements, in accordance with younger adults’ neural capacity. Additionally, in YA, HbO and HbR signals were both coherent and reliable indicators of brain activity that correlated with improved accuracy in the single task. Note that Meidenbauer et al. [40] only reported HbR values and their relationship with accuracy (not RT) and did not find any significant relationships between HbO and behaviour, suggesting that the slower HbR signal may better capture task-related metabolic changes over time, unlike the larger, more immediate HbO signal, which is more sensitive to blood flow changes [45]. In contrast, our findings showed that younger adults exhibited significant brain activity-performance relationships with both HbO and HbR signals (see relationships with error rates, Table 1). This indicates the need for further investigation to understand whether load-dependent effects are driven by one signal or process over the other (i.e., metabolic demands vs. blood flow changes) or both.

Interestingly, PLS analysis of YA’s dual task did not reveal any significant associations between HbR or HbO and behaviour measures (RT and accuracy). A null relationship may indicate that the cognitive demands of managing two stimuli simultaneously altered the relationship between brain activity and performance. In this case, increasing neural activity in frontal regions may not be sufficient to improve performance or may even be insufficient due to the increased complexity of the task. Thus, the absence of the relationship in YA’s suggests that managing dual tasks may require different cognitive strategies or neural mechanisms that do not rely solely on increased frontal activity to enhance performance [68]. This highlights the importance of considering task-specific factors and cognitive demands when interpreting relationships between brain activity and performance.

In Ranchod et al. [32] we suggested that YA’s decreased brain activity in the dual task relative to the single task may have indicated neural disengagement or lack of cognitive effort [48, 54], rather than an indication of task efficiency. When participants disengage or "give up", their neural activity may decrease, leading to a lack of association between brain activity and performance in our PLS. The decision to disengage from the task could result from various factors, such as perceived task difficulty, lack of motivation, or fatigue. It is also possible that the cognitive demands of the dual task exceeded the participants’ cognitive capacity, leading to this task disengagement [35, 48]. This would suggest that the relationship between brain activity and performance is influenced not only by cognitive processes but also by motivational and affective factors [54, 69, 70]. Understanding the interplay between these factors would be essential for interpreting load-dependent brain-behaviour relationships accurately. For example, Jaeggi et al. [54, 68] performed post single and dual task interviews to examine motivational factors and found differences in brain activity associated with effort and Zhuang et al. [56] implemented scale invariance and PLS methods to fNIRS data collected over increased cognitive load (N-back) and identified load-dependent brain activity changes. Combining these methods may provide insight about whether perceived effort or fatigue explain addition (e.g., compensatory brain activity) or attenuation of brain activity.

Our results also highlighted distinct age-group relationships between brain-behaviour across task loads. Specifically, we found OA’s single task HbO unrelated with task performance measures, but HbR was negatively correlated with error rate in this task, contrasting YA’s positive relationship between HbR and error rate. It should also be noted that the HbR by RT relationships, although not significant (*p* = 0.055) also supported this pattern of response in OA and YA. This suggests that in OA, increased brain activity, indicated by reduced HbR concentration, may be associated with poorer performance, i.e., trend for longer RT and greater error rates, contrary to YA. HbR but no HbO association likely demonstrates a less robust relationship between brain activity and behavior in the single task. In the dual task, OA exhibited significant relationships with performance, indicating that as brain activity increased, error rates decreased; however, this relationship was observed in HbO and not HbR, although HbR appeared to have the positive relationship trend that would support this dual task observation. Additionally, OA’s strong trend for longer RTs was associated with HbR but not HbO, suggesting that increased brain activity (or decreases in HbR) led to longer RTs, as seen in the single task for this age-group, but contrary to YA.

Overall, the complexity of the task appears to have influenced OA’s pattern of brain activation and its relationship with performance. OA’s relationship between brain activity and RT, mostly involving channels in the right hemisphere, may indicate a speed-accuracy trade-off where OA slow down either as a strategy to maximize or maintain accuracy or because of structural and physiological changes to the brain, particularly in connectivity between PFC areas such as the pre-SMA with the striatum [71]. Thus, this speed for accuracy trade-off may act as a compensatory mechanism of age-related changes, observed in our tasks. However, to directly assess whether this speed-accuracy trade-off is occurring, follow-up studies that implement varying time limits on stimulus responses whilst also implementing mathematical models that account for the speed-accuracy trade-off would be needed [71]. Furthermore, given the variability of the HbR signal, more research is likely needed to better understand the effects of brain activity patterns on RT and their impact on performance accuracy.

The error rate and brain activity relationship in our single task showed that older adults may not need or may not be able to effectively use additional brain resources (HbO) to improve performance. The lack of a HbO-error rate relationship may suggest inefficiencies or heterogeneity in brain activation patterns that do not contribute positively to performance in the low load single condition, in accordance with the neural inefficiency theory [5, 15, 16]. Alternatively, the absence of a significant relationship may reveal heterogeneity in the cognitive strategies and/or brain function implemented by OA, leading to a more diffuse pattern of brain activity within this single task. However, when examining participants’ single task error rates (*M* = 21.86, SD = 17.88) the lack of HbO association in the single task could be explained by relatively good and narrow range of scores (vs. Dual task, *M* = 32.28, SD = 20.37, Ranchod et al. [32]) limiting the ability to find relationships.

In contrast to the single task, enhanced brain activity supported OA’s performance in the more cognitively demanding dual task, as seen in the significant HbO relationship, and confirming previous findings in Ranchod et al. [32]. The dual task’s increased cognitive load may therefore require and benefit from the recruitment of additional brain activity, indicating compensatory mechanisms [10, 17]. Furthermore, the presence of a significant HbR relationship in the single task but not in the dual task revealed that the brain activity measured by HbR may be less reliable or more variable in its impact on performance. In the single task, where the cognitive demand is lower, the fluctuations in HbR might reflect a less consistent or less beneficial pattern of brain activity. In contrast, in the dual task, the more reliable HbO relationship might dominate, making HbR less relevant.

The differences in relationships between brain activity and performance across task load conditions highlight the impact of task complexity in understanding cognitive aging. The findings suggest that older adults can mobilize and utilize additional brain resources (indicated by HbO) to support performance in more cognitively demanding tasks, as a compensatory mechanism afforded by neural capacity [17]. Thus, no significant HbO-error rate relationship and a negative HbR-error rate relationship in our single task would likely suggest alternative explanations over inefficiency (e.g., small score variability or different strategies). This indicates that in OA, the relationship between brain activity and performance is less consistent, with only one type of signal (HbO or HbR) correlating with performance depending on the task.

The PLS analysis revealed distinct load-dependent brain activity patterns between the age-groups not identifiable in the previous univariate analysis [32], that further and more directly showed that OA can recruit additional brain activity at higher task complexities, indicating compensation as reported in Ranchod et al. [32], but the efficiency and effectiveness of this recruitment can vary depending on the task demands and individual differences within the population.

The study’s limitations are thoroughly discussed in Ranchod et al. [32]. It is worth adding that, like many fNIRS studies, our array was restricted to frontal lobe which may limit our understanding not only of the brain areas involved in our single and dual tasks but also our understanding of age-related brain activity patterns. Related to the present study’s PLS analysis specifically, we cannot rule out other potential effects that could explain our null or significant relationships, such as age-related differences in blood flow regulation, nor other potential mechanisms explaining brain-behaviour relationships like neural inefficiency or dedifferentiation, given that no causal effects were identified. Furthermore, these brain activity mechanisms may not be mutually exclusive [10].

Although we used the most robust methods to eliminate noise [53, 72], PLS analysis is sensitive to the quality and variability of the input data and any noise or inconsistencies in the fNIRS data can impact the robustness of the findings. As Meidenbauer et al. [40] suggested, the HbO and HbR signals differ in their underlying physiological mechanism (i.e., oxygen metabolism vs. blood flow for HbR and HbO respectively) [45] and may exhibit age-related differences that have not been thoroughly defined. Furthermore, one of our analyses displayed a strong trend (HbR by RT *p* = 0.05) and may be interpreted with caution. However, the relationships within this analysis displayed similar magnitudes and confidence intervals to those all other PLS analyses. In addition, Batterham and Hopkins [73] and others advocated by learned societies [74] emphasize not only relying on p-values but focusing on effect size and confidence intervals, as we have done here.

Finally, although none of our participants had hearing aids or reported hearing deficits, had normal MOCA scores, which tests for attention to auditory letters, and we made sure that our participants were able to hear the auditory stimuli in the practice session, we did not formally assessed hearing. Thus, it is possible that listening effort could have impacted dual-task performance and brain activation, leading to different brain activity patterns between younger and older participants [75]. Older participants, even with mild hearing loss, may require more effort to hear the stimuli resulting in increased neural recruitment which allowed them to perform well behaviourally.

## Conclusion

The present study examined load-dependent brain and behaviour relationships in young and older adults using PLS on fNIRS brain imaging data and measures of performance across a single and a dual 2-back task. The PLS analysis revealed distinct age-related patterns in neural activation and their association with performance. Importantly, older adults demonstrated compensatory brain activity, with increased HbO linked to better performance under higher cognitive load in the dual task. The PLS analysis accounted for individual variability more effectively than the previously implemented univariate, group-averaged approach, providing a more precise identification of brain-behavior relationships and deeper insights into the neural processes underlying cognitive aging. These findings emphasize the importance of considering task complexity and individual variability when interpreting age-related changes in brain activity. They contribute to a better understanding of cognitive aging, showing the variability in compensatory mechanisms and the advantages of robust, individualized analytical approaches in aging research.

## Supporting information

S1 Fig Scatterplot of all channels with BSR > 2 for the HbO by RT relationship for all groups and task levels. (PDF)

S2 Fig Scatterplot of all channels with BSR > 2 for the HbR by RT relationship for all groups and task levels. (PDF)

S3 Fig Scatterplot of all channels with BSR > 2 for the HbO by ER relationship for all groups and task levels. (PDF)

S4 Fig Scatterplot of all channels with BSR > 2 for the HbR by ER relationship for all groups and task levels. (PDF)
