# Supplementary figures and images for: Using multivariate partial least squares on fNIRS data to examine load-dependent brain-behaviour relationships in aging
Source: PLoS One. 2024 Oct 14;19(10):e0312109. doi: 10.1371/journal.pone.0312109 (PMC11472942; doi:10.1371/journal.pone.0312109)

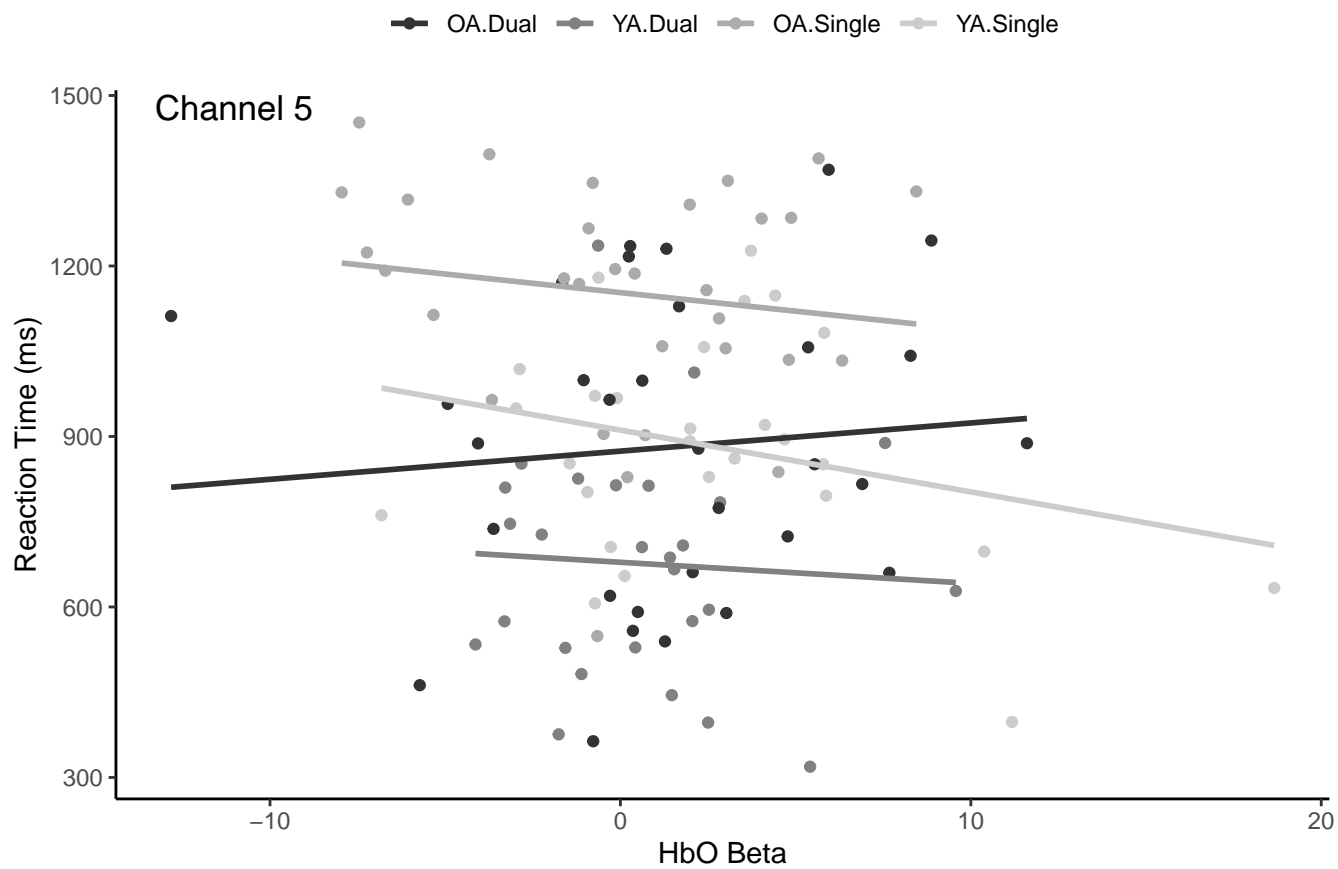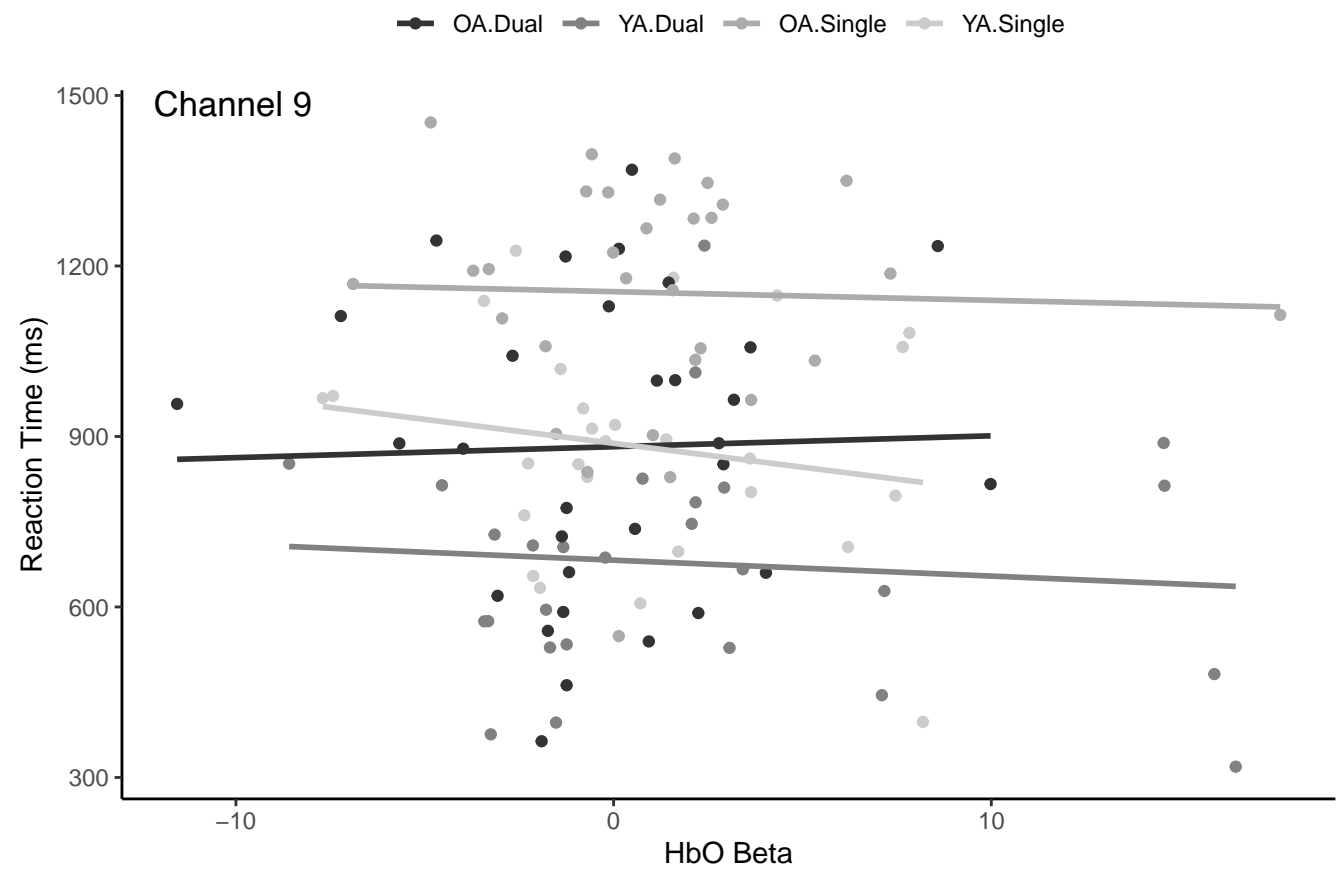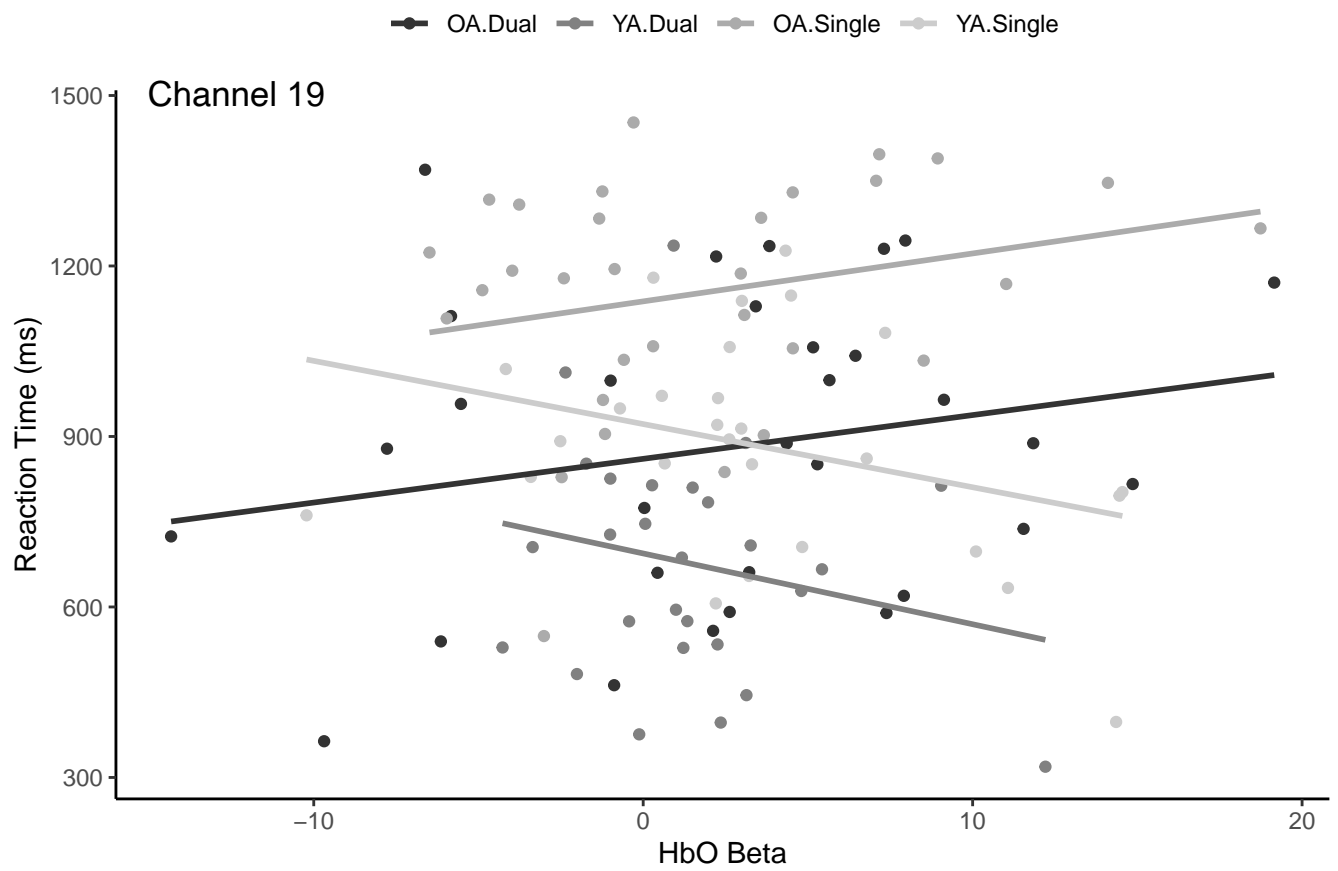

Supplement: S1 Fig — (PDF) [file pone.0312109.s001.pdf]

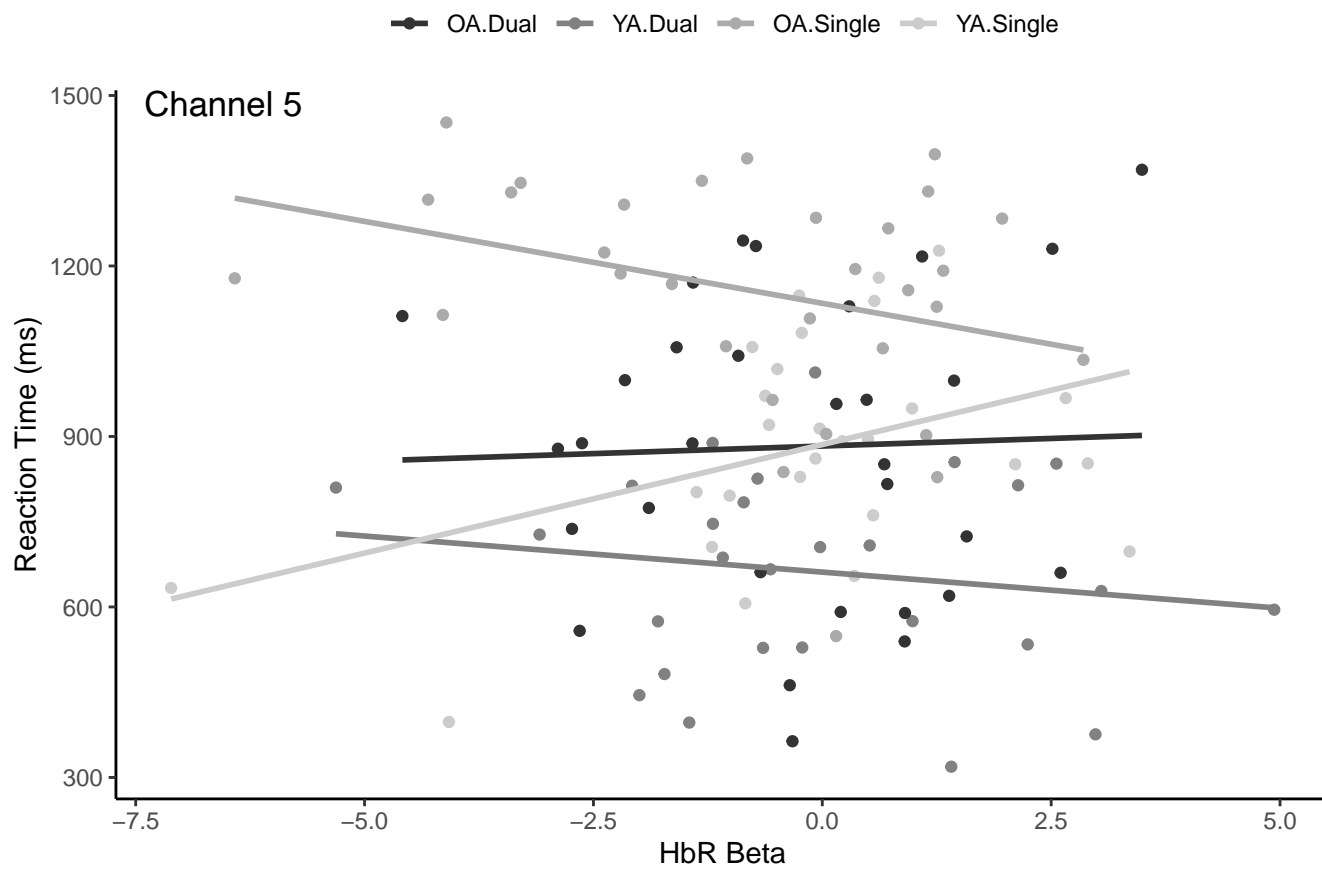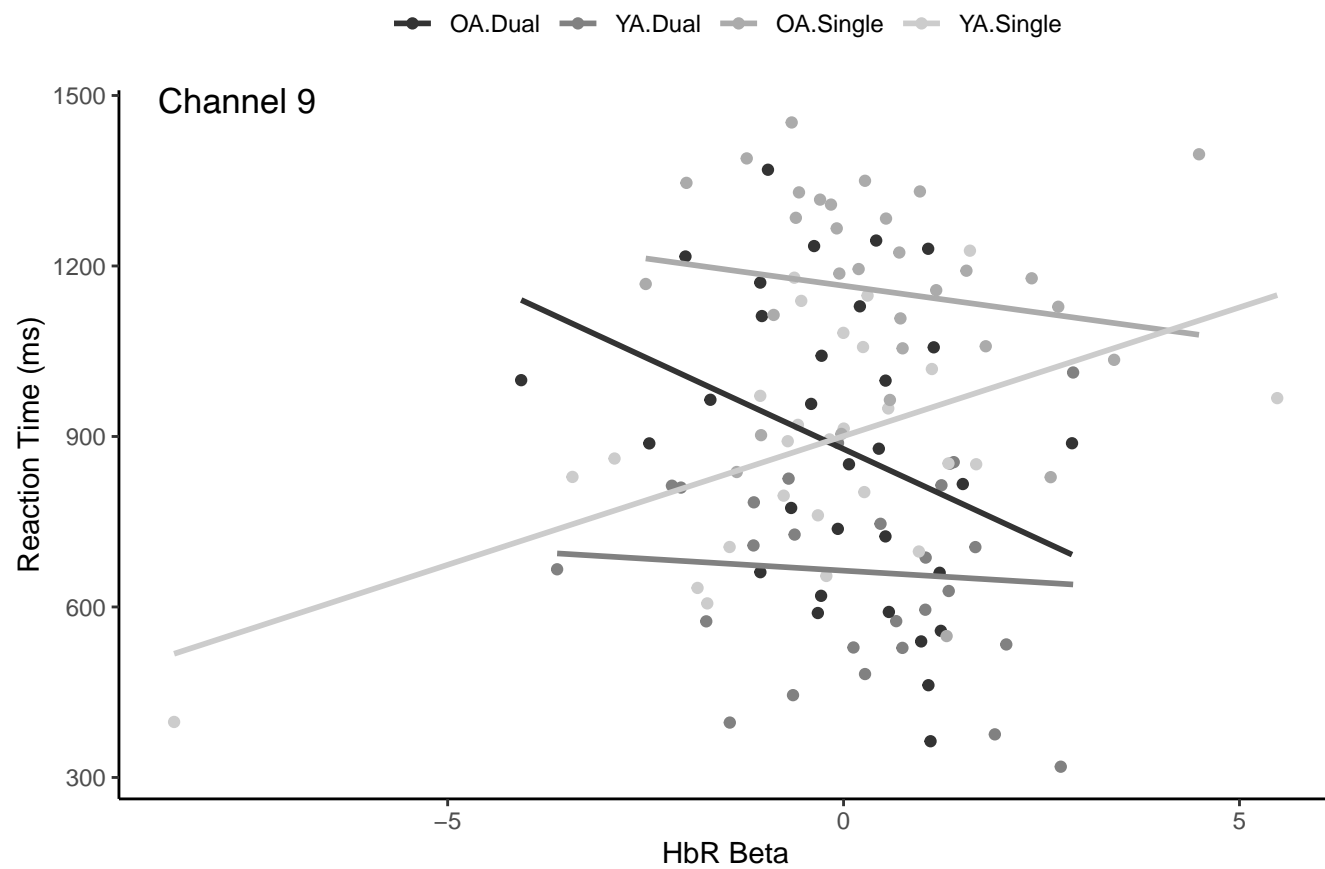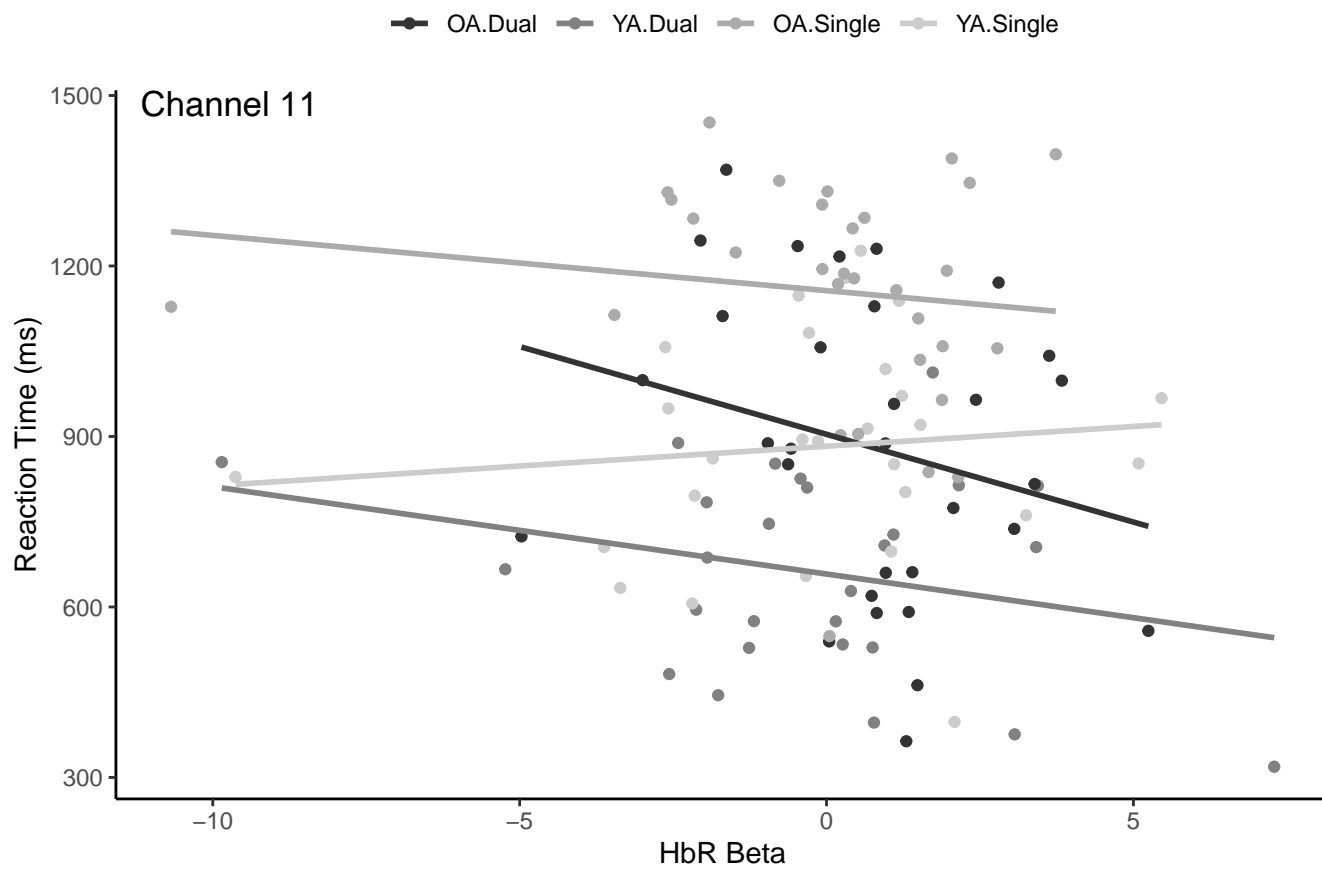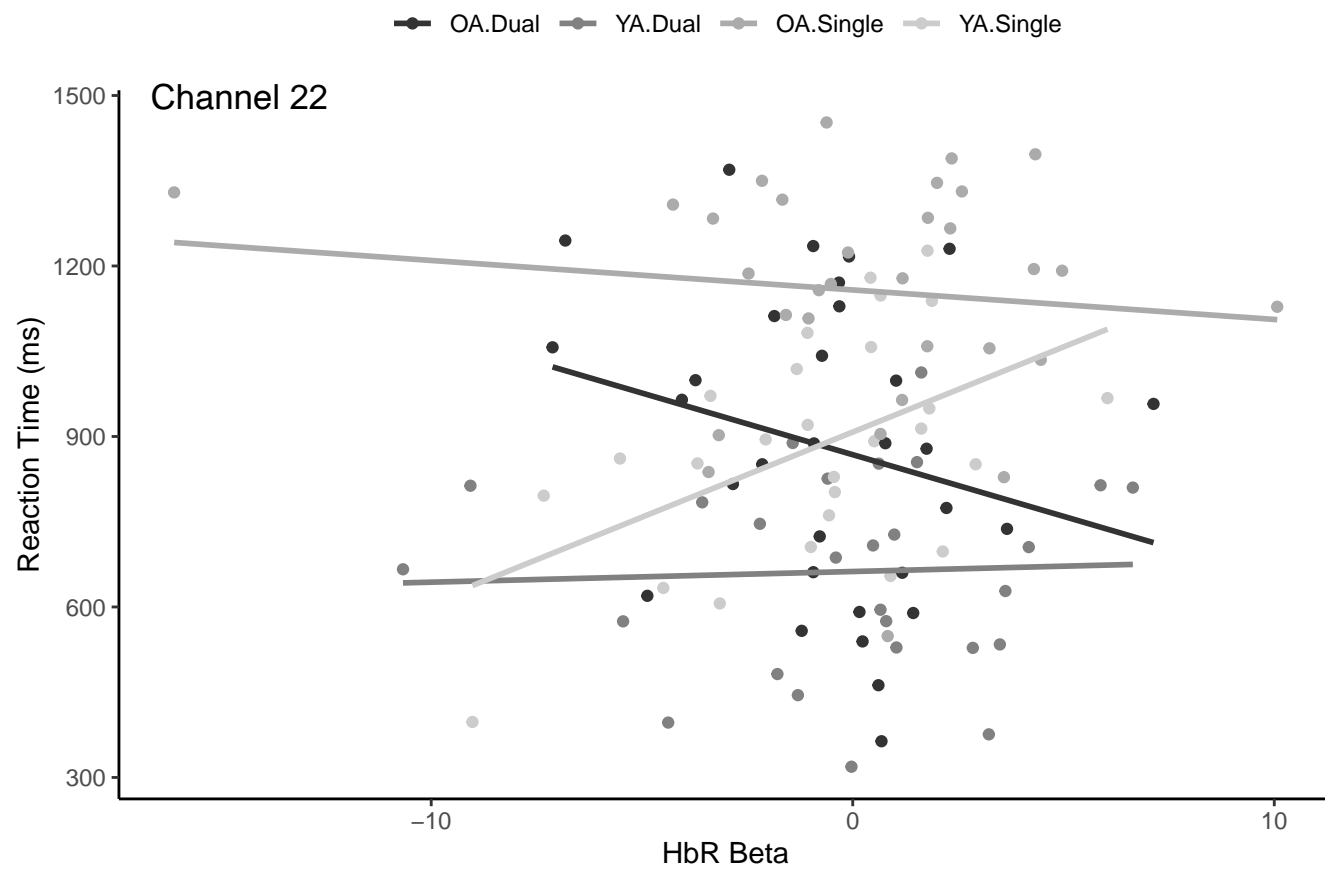

Supplement: S2 Fig — (PDF) [file pone.0312109.s002.pdf]

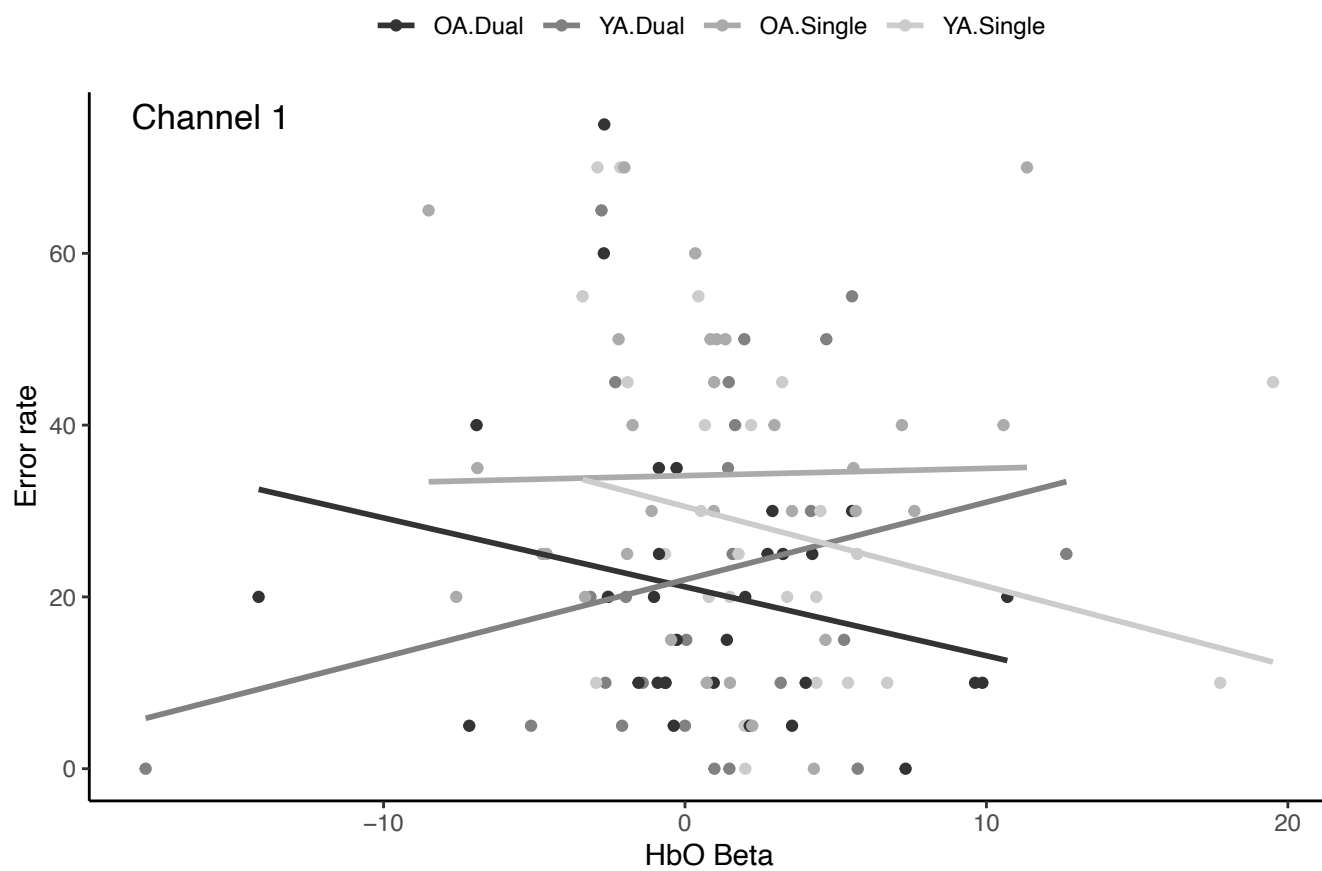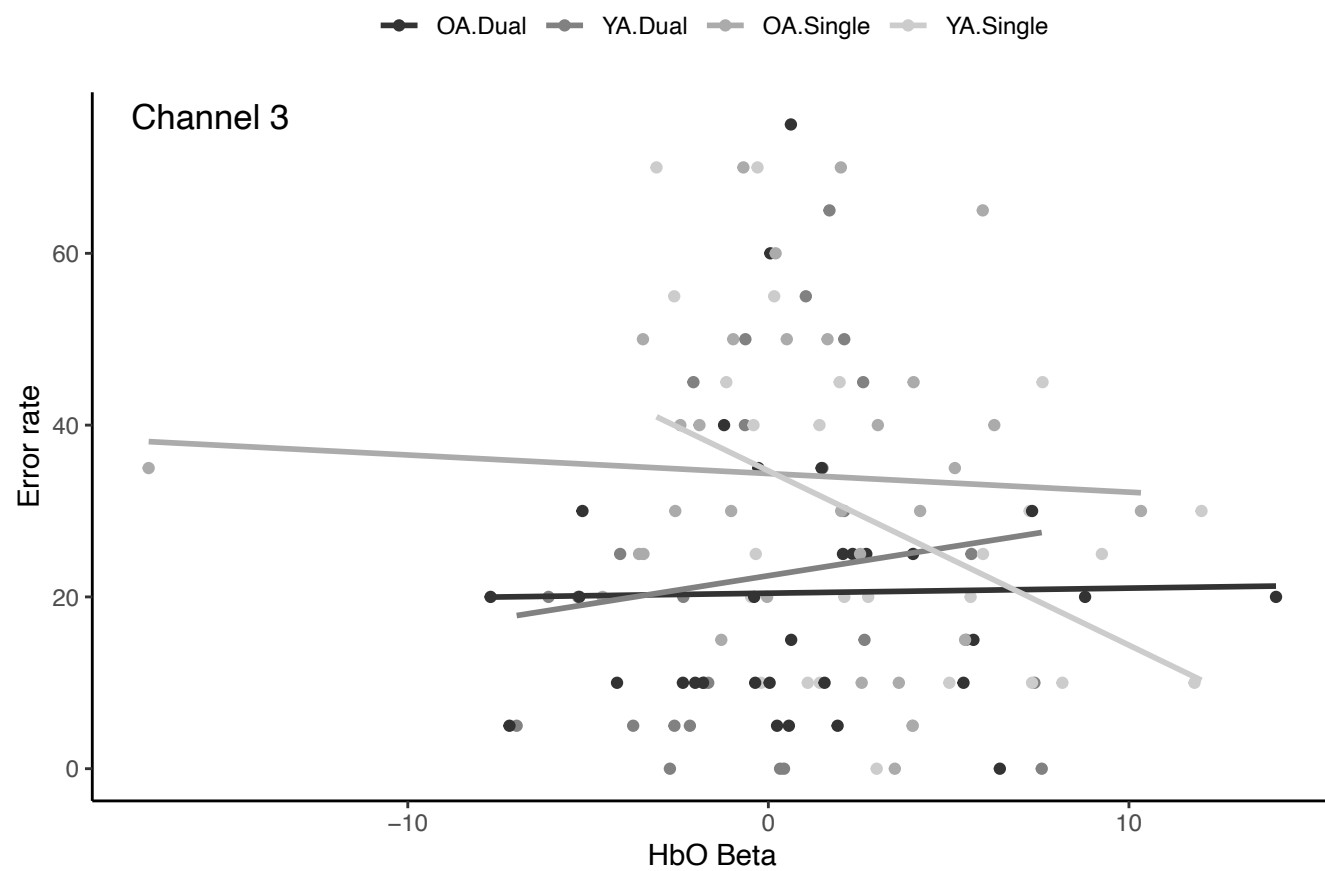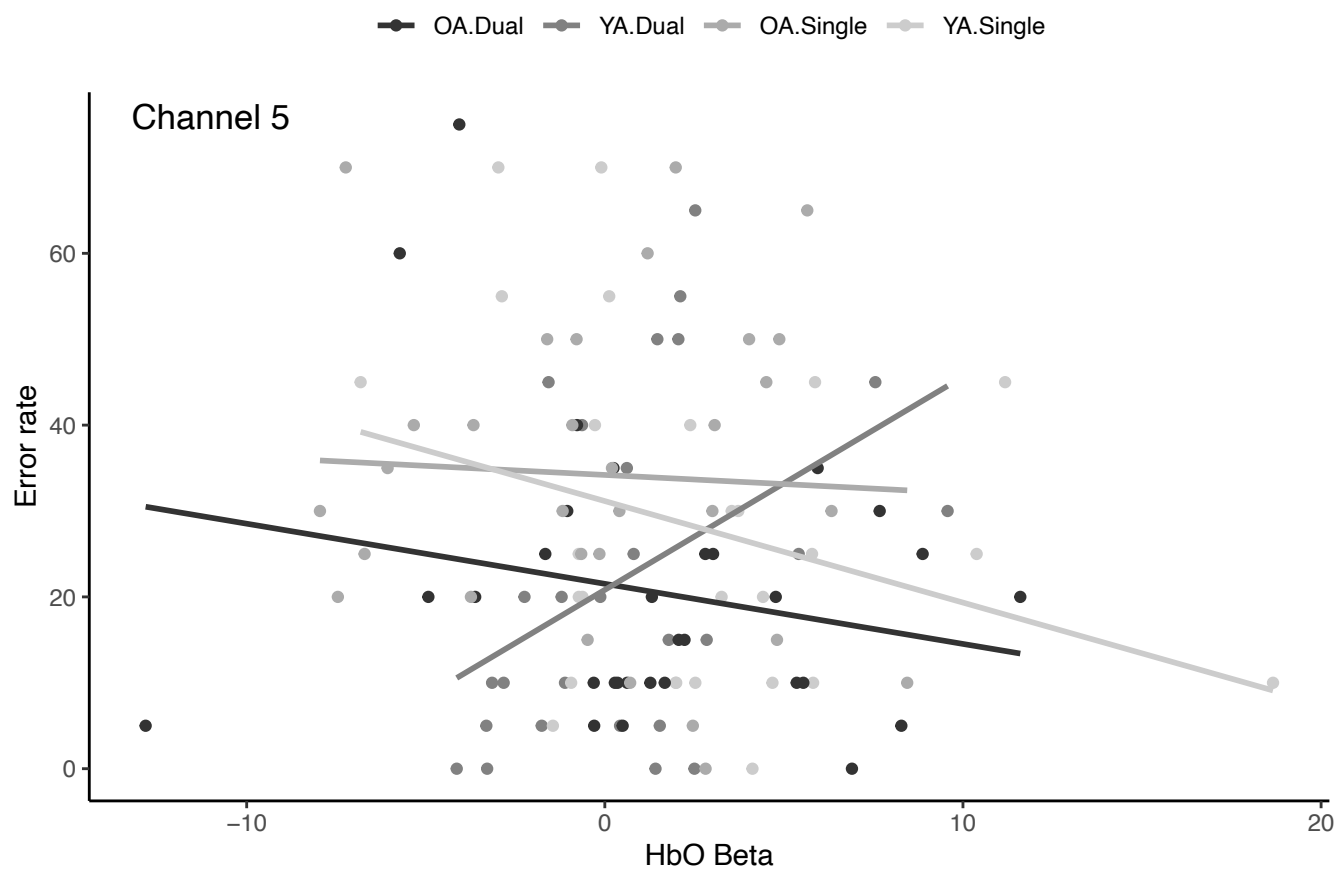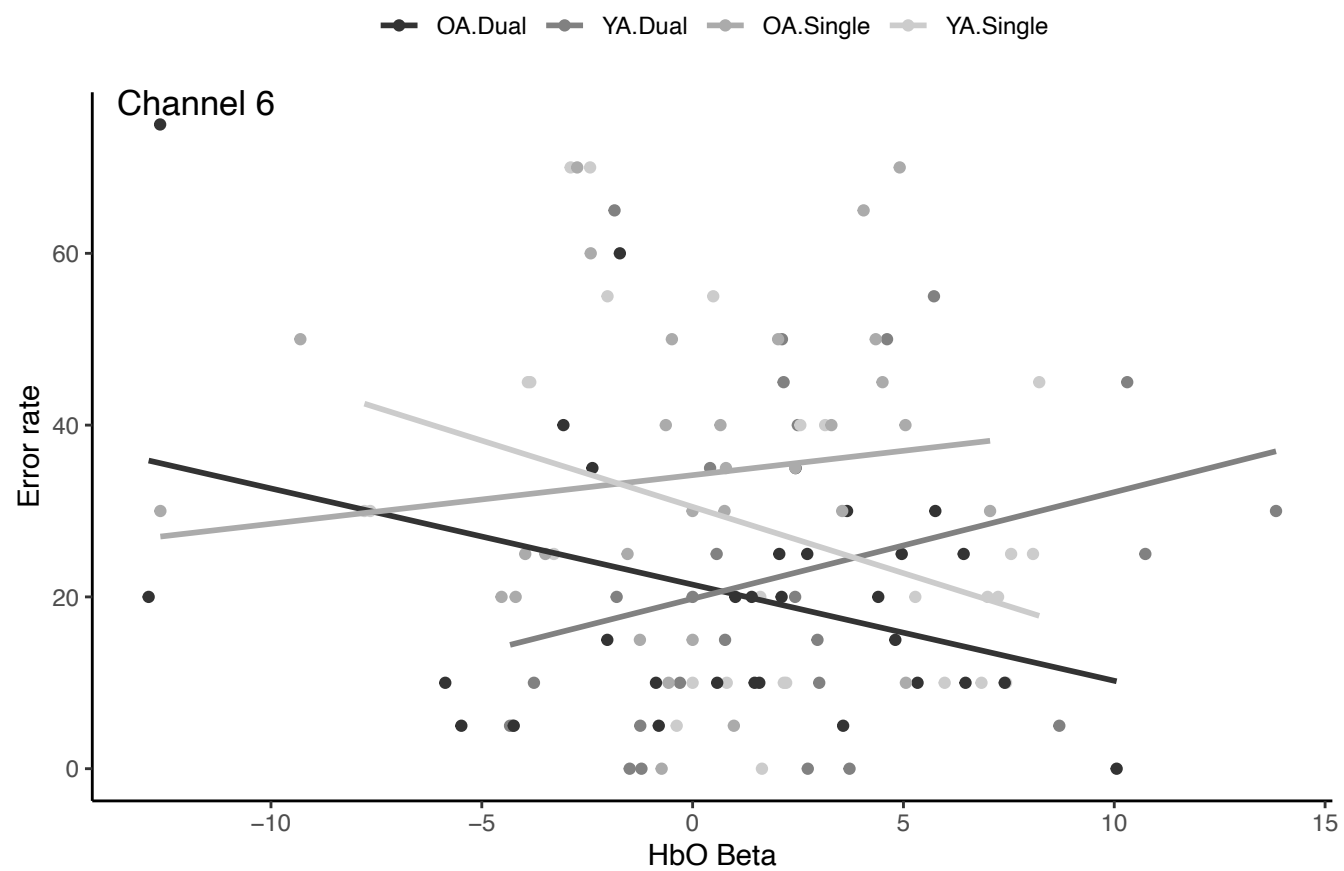

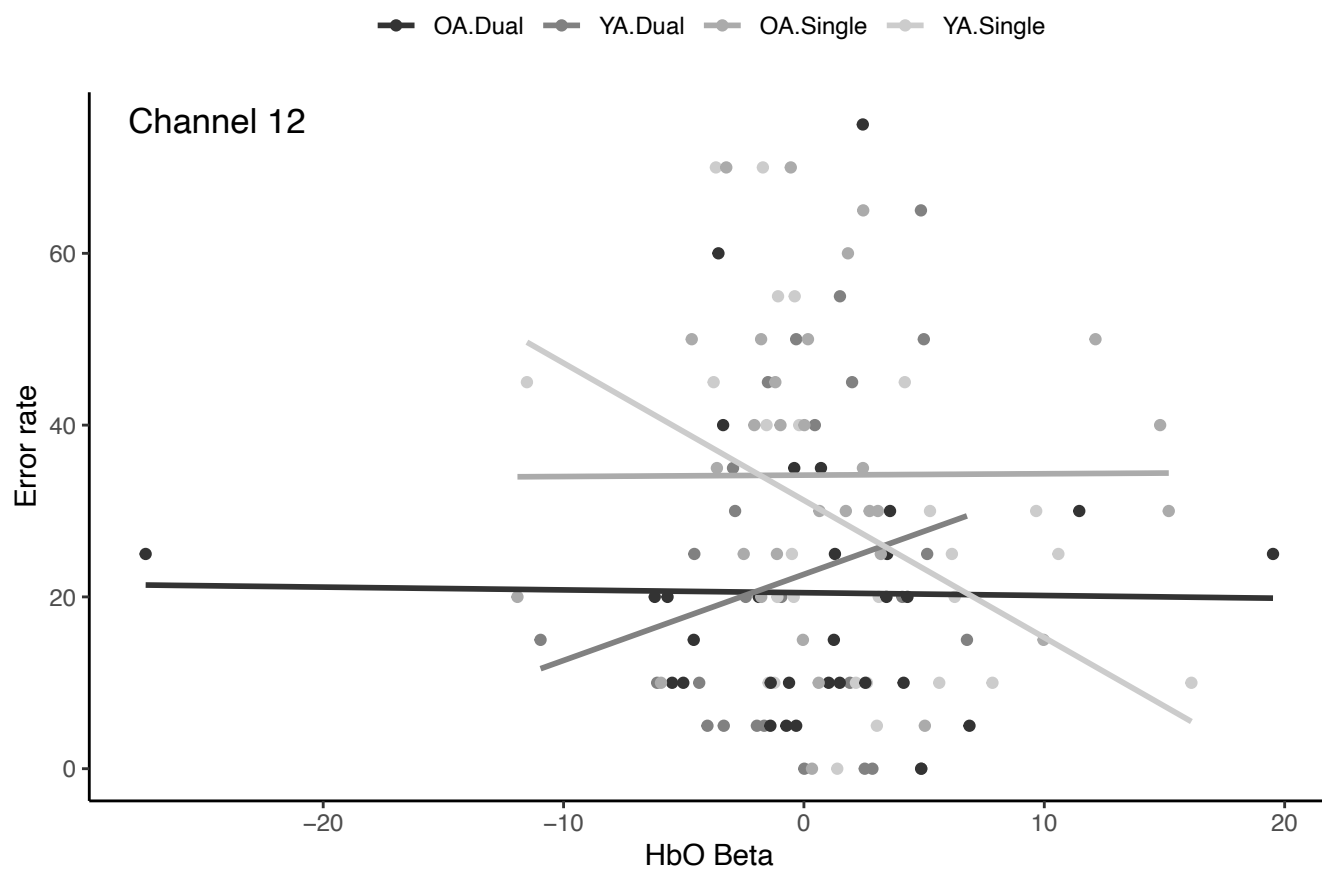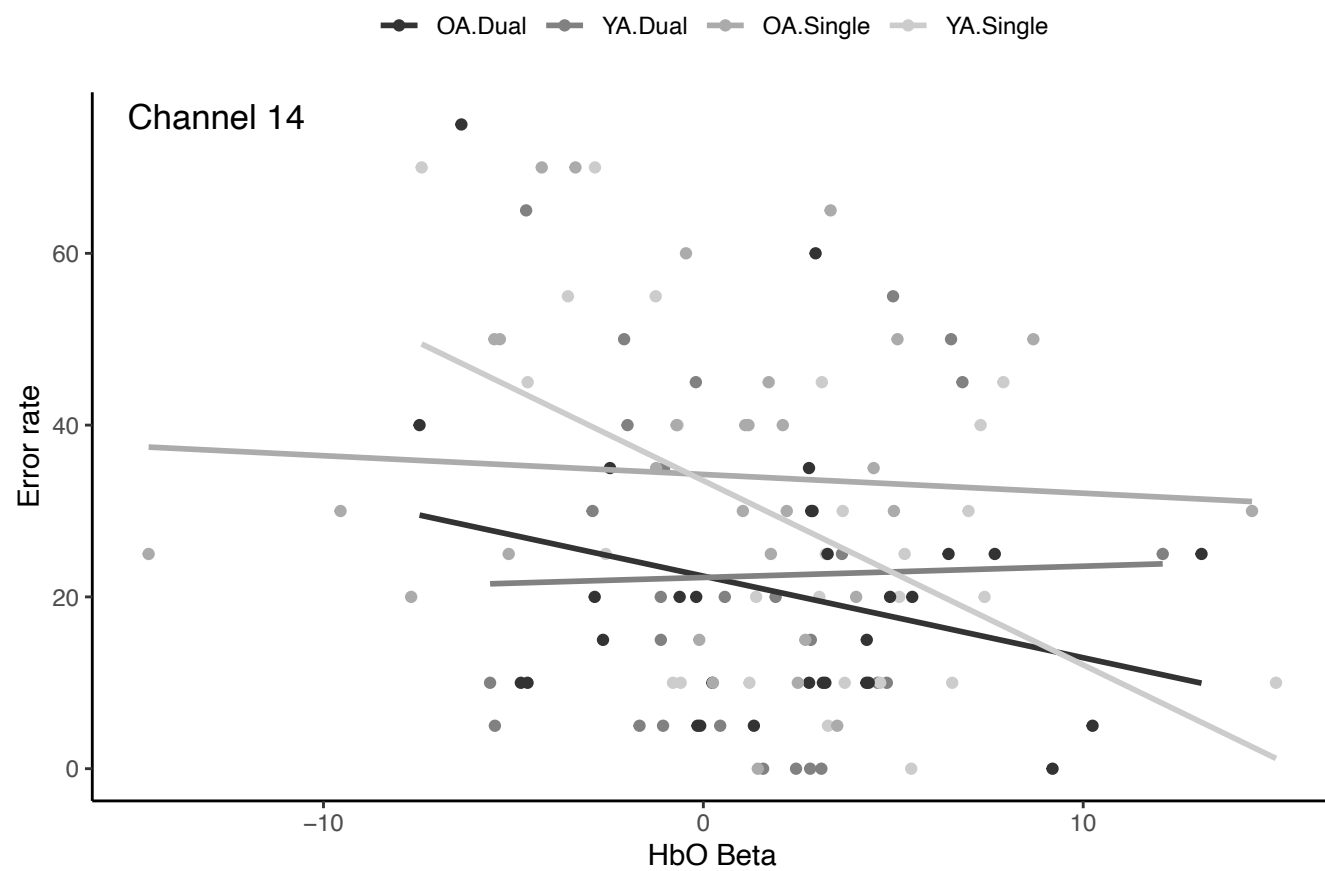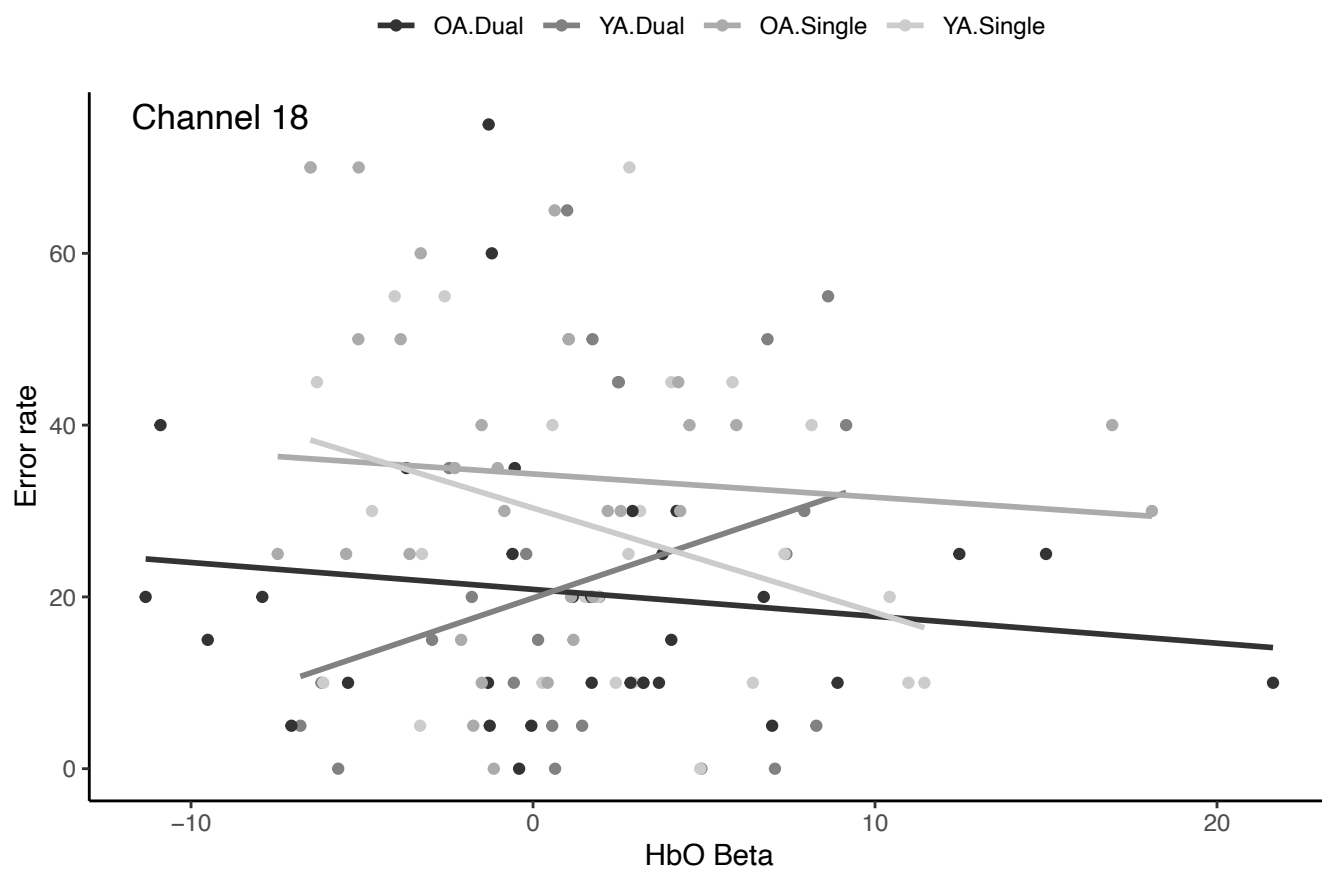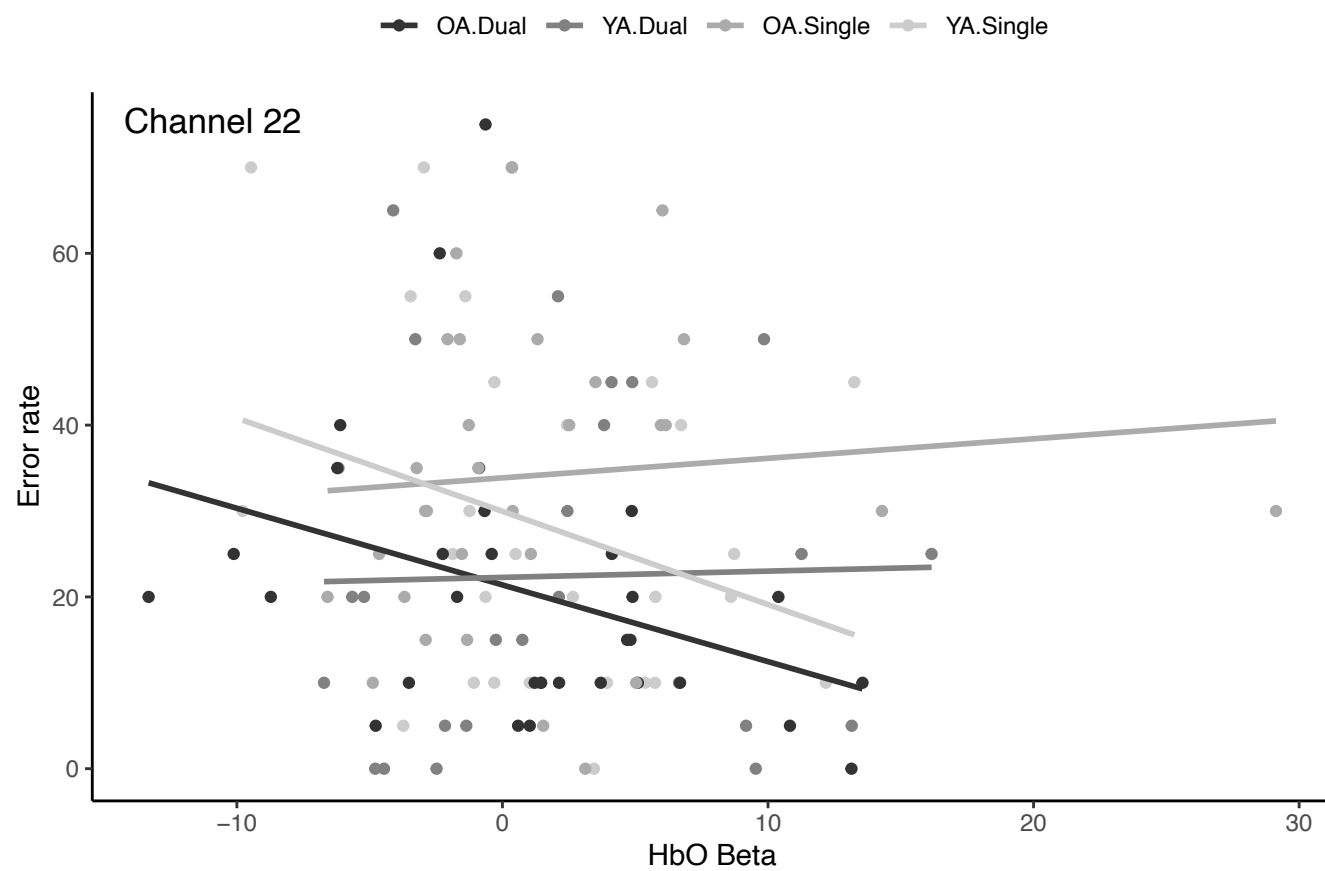

Supplement: S3 Fig — (PDF) [file pone.0312109.s003.pdf]

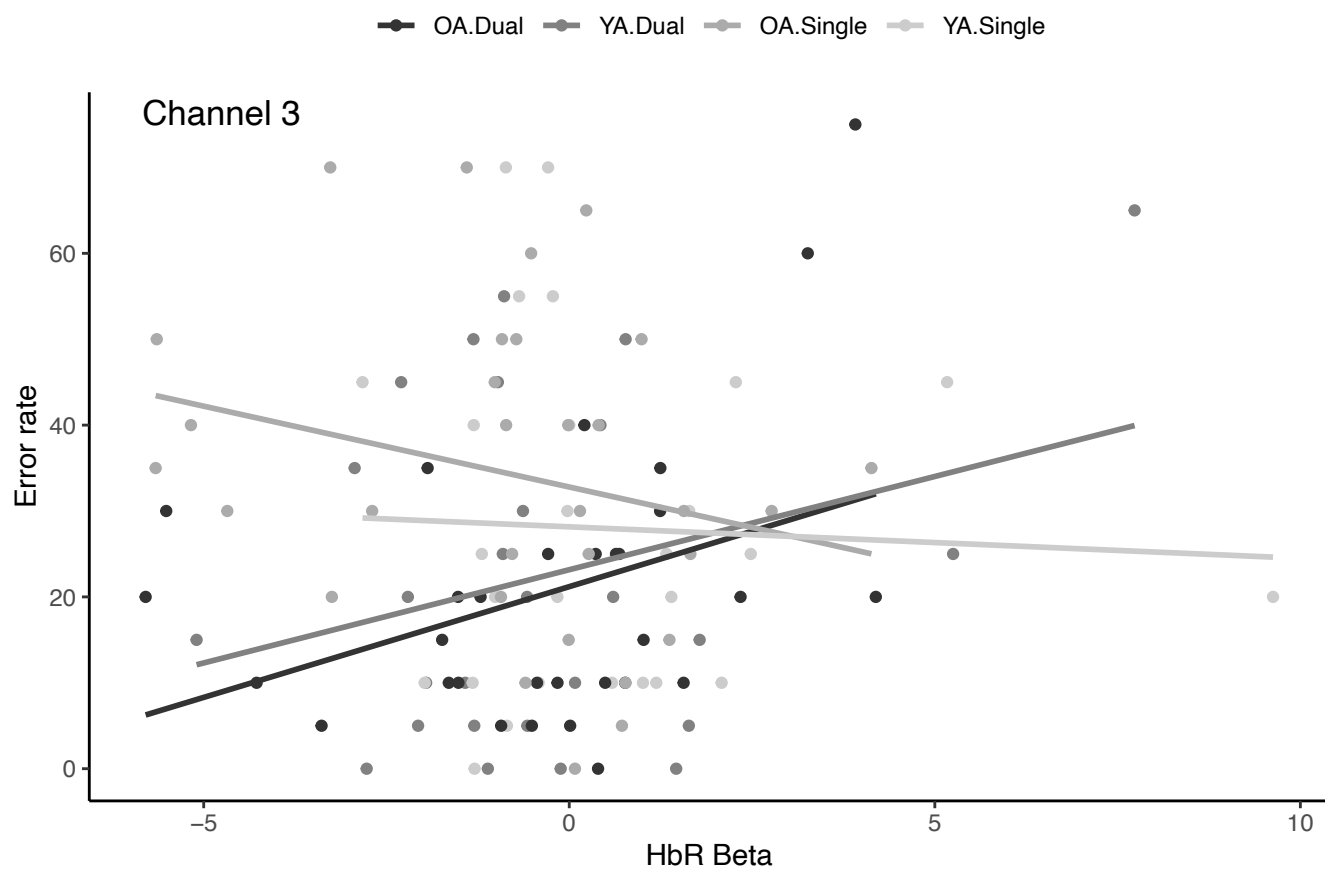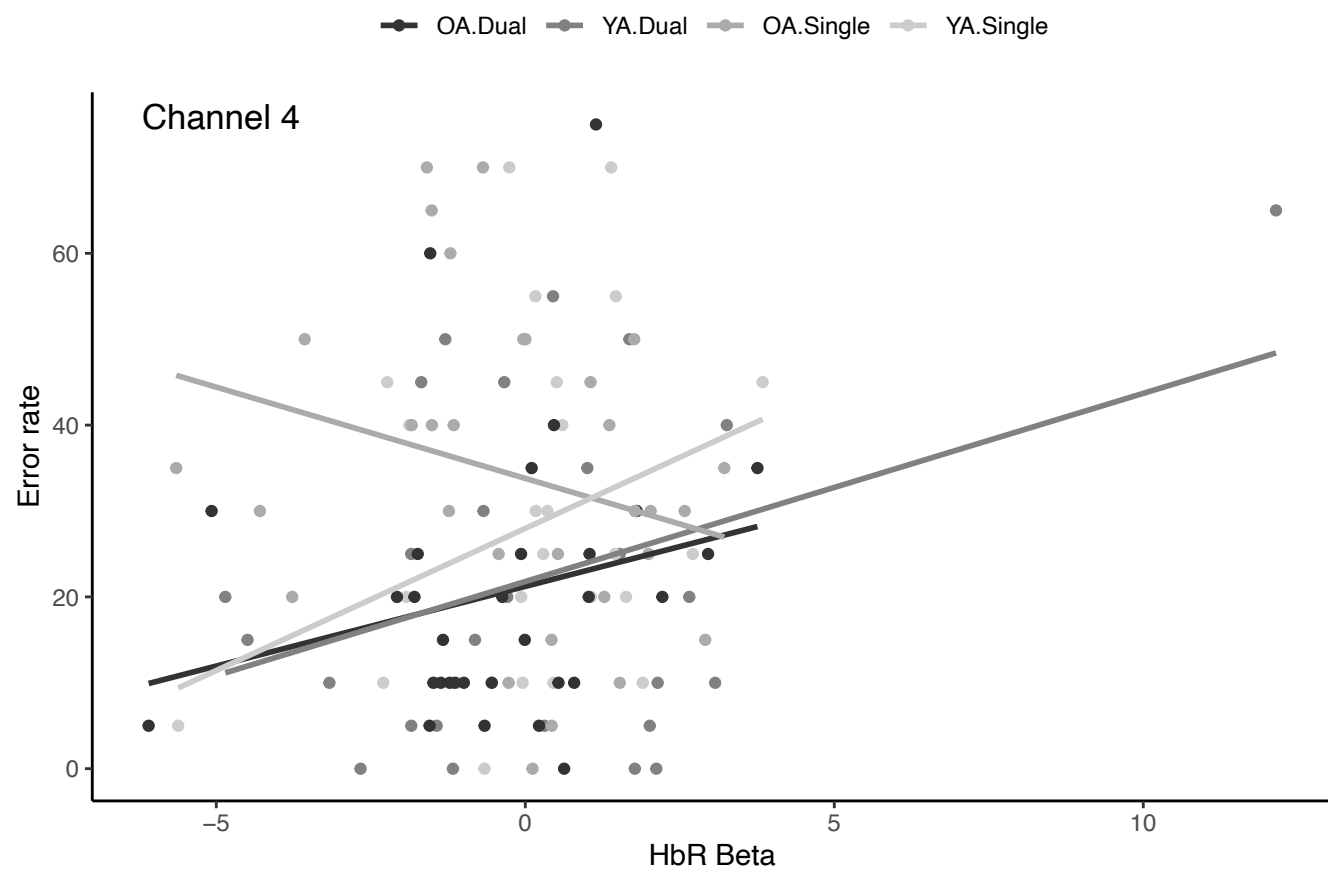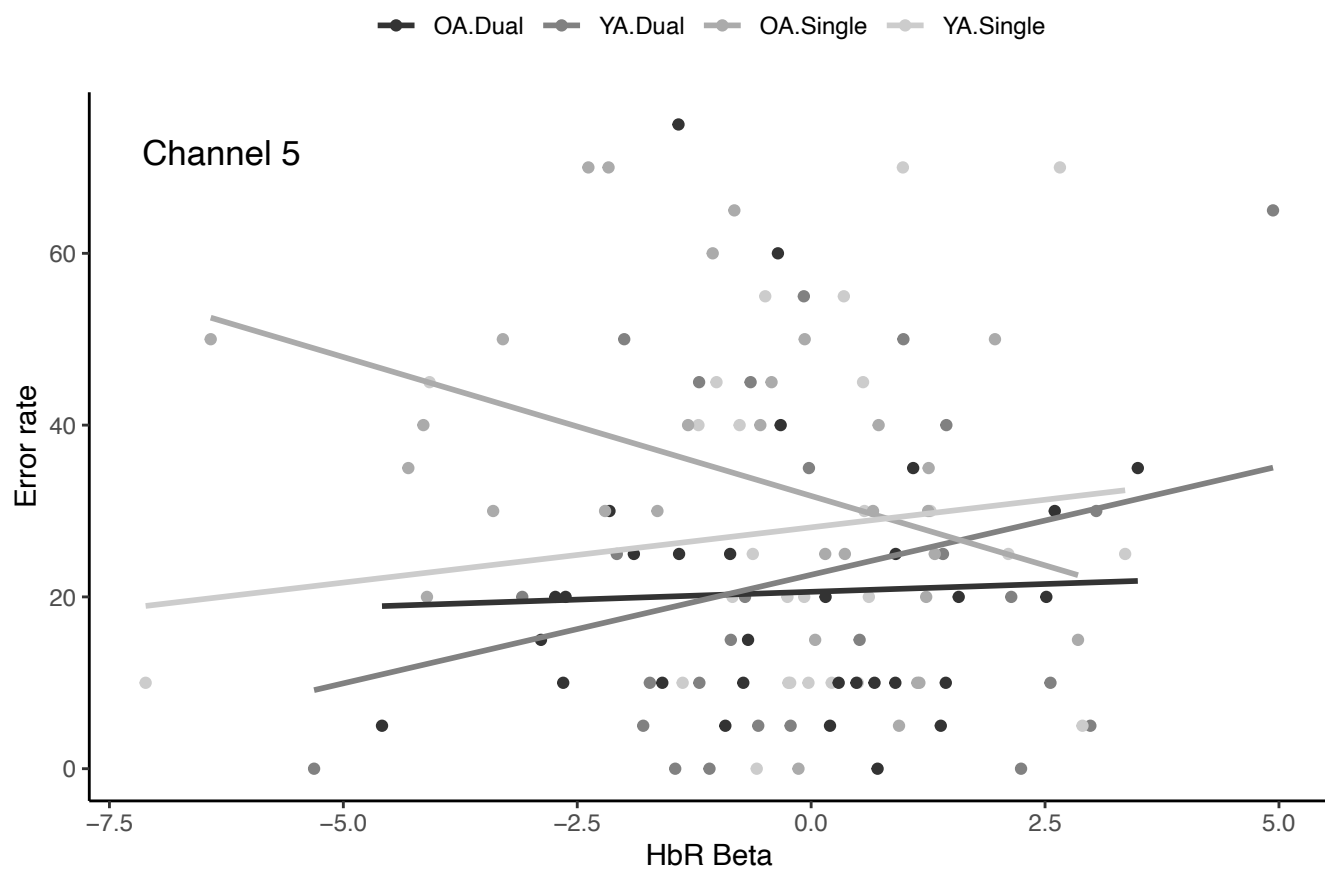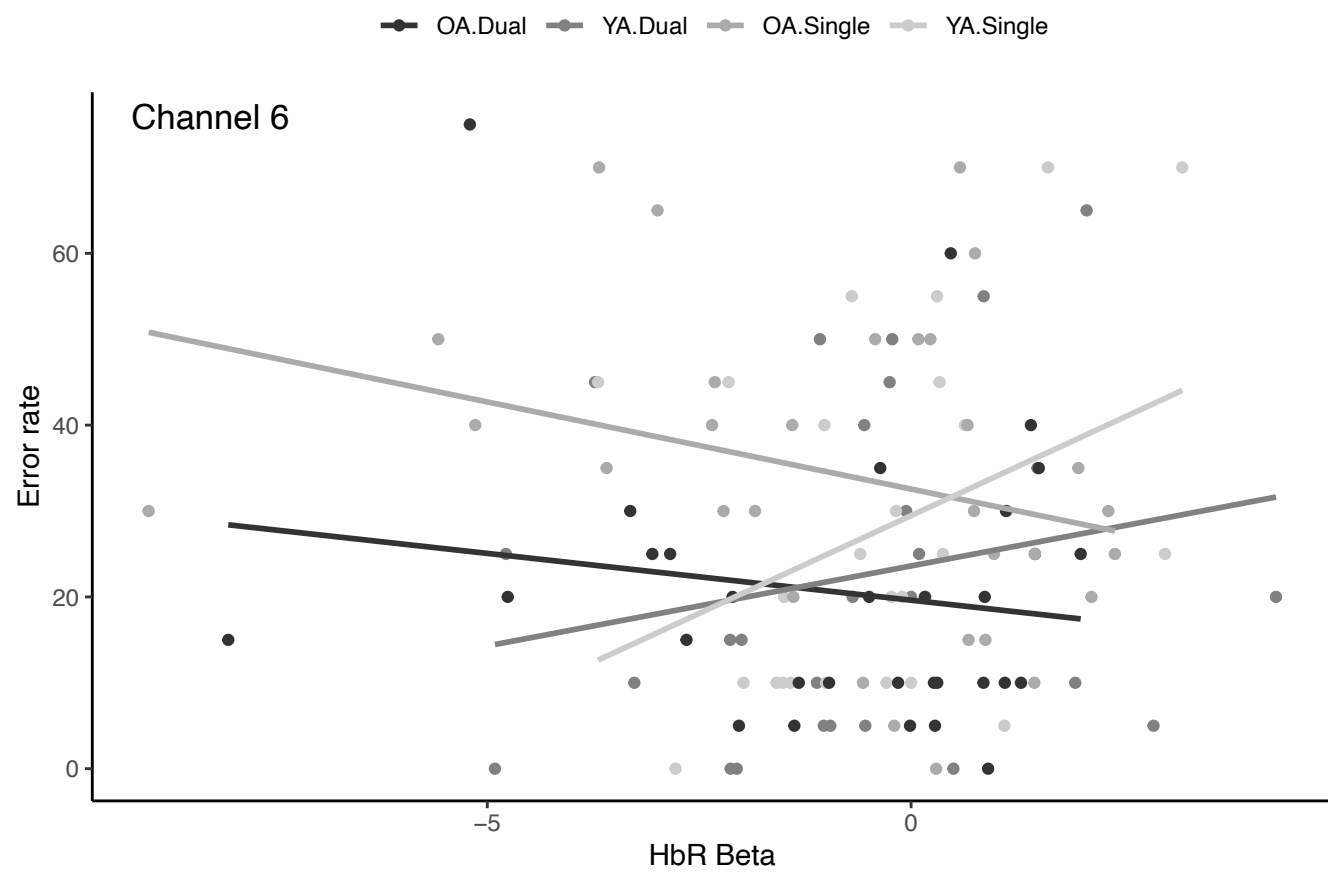

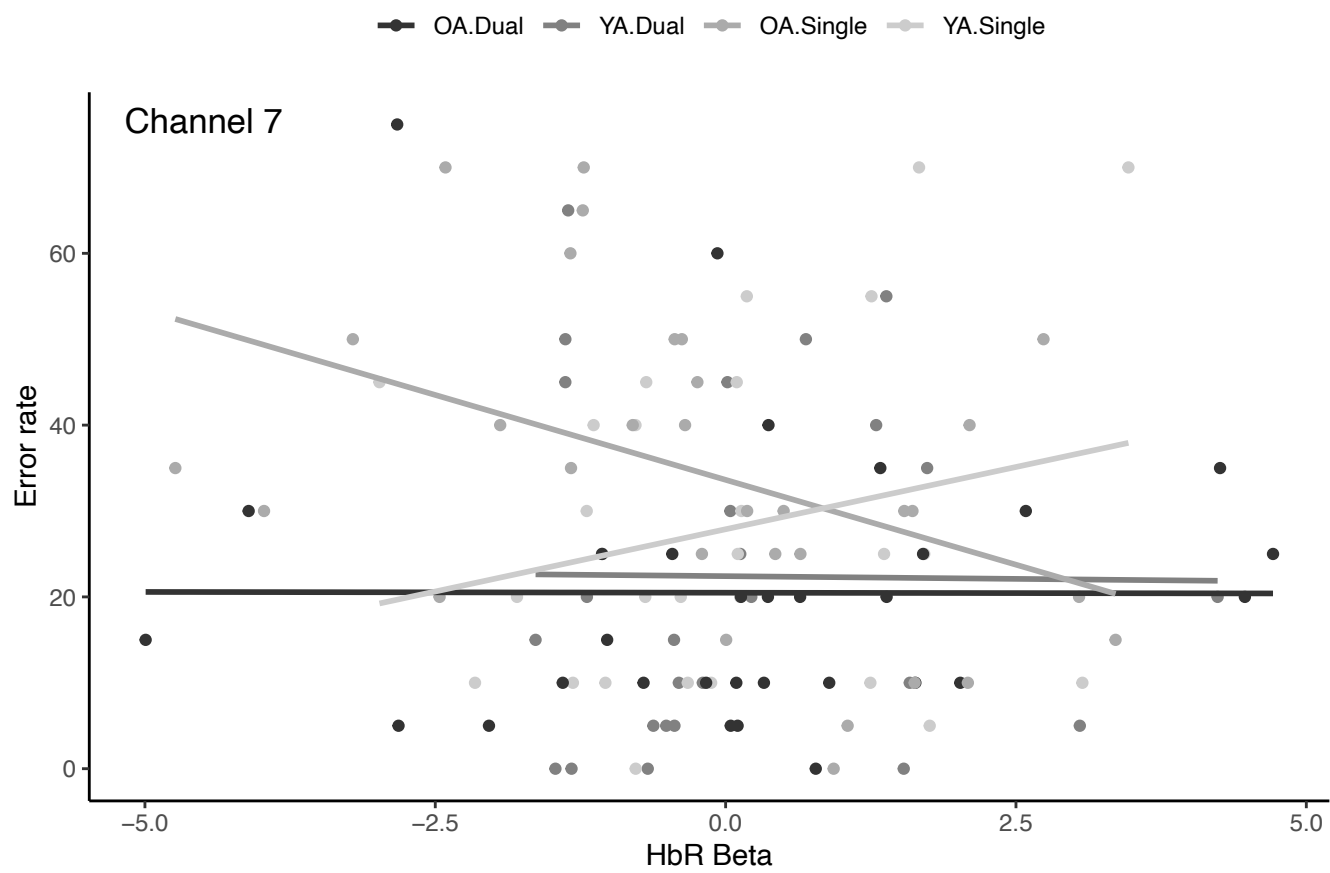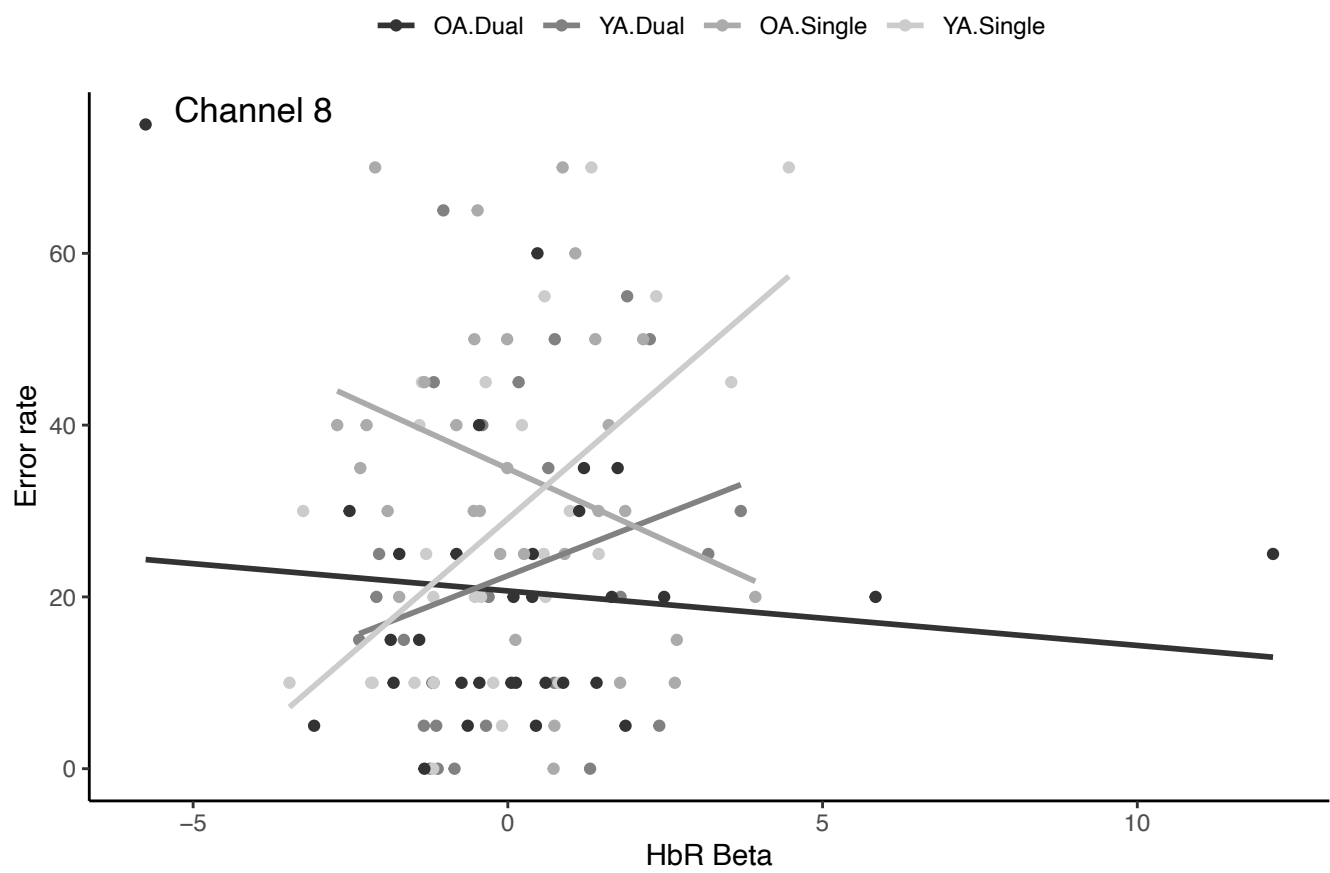

Supplement: S4 Fig — (PDF) [file pone.0312109.s004.pdf]
